# Nanoparticles design considerations to co-deliver nucleic acids and anti-cancer drugs for chemoresistance reversal

**DOI:** 10.1016/j.ijpx.2022.100126

**Published:** 2022-09-06

**Authors:** Sahar Eljack, Stephanie David, Areeg Faggad, Igor Chourpa, Emilie Allard-Vannier

**Affiliations:** a EA 6295 Nanomédicaments et Nanosondes, Faculté de Pharmacie, Université de Tours, Tours, France; b Department of Pharmaceutics, Faculty of Pharmacy, University of Gezira, Wadmedani, Sudan; c Department of Molecular Biology, National Cancer Institute, University of Gezira (NCI-UG), Wadmedani, Sudan

**Keywords:** Combination therapy, Nucleic acids, Anti-cancer drugs, Chemoresistance reversal, Nanoparticles, **5-FU**, 5-Flurouracil, **ABCB**, ATP Binding Cassette Subfamily B Member, **AIF**, Apoptosis-inducing factor, **AKT**, Serine/threonine kinase, **ASGPR**, The asialoglycoprotein receptor, **ASO**, Antisense oligonucleotides, **Bak**, Bcl2-antagonist/killer, **Bax**, Bcl-2-associated X protein apoptotic activator, **BBB**, Blood-brain barrier, **Bcl-2**, B-cell lymphoma 2, **Bcl-xl**, B-cell lymphoma-extra large, **BCRP**, Breast cancer-resistant protein, **CMD**, Carboxymethyl dextran, **CAV-1**, Caveolin 1, **CDK**, Cyclin-dependent kinase, **ChNPs**, Chitosan nanoparticles, **CI**, Combination index, **CisPt**, Cisplatin, **CPT**, Camptothecin, **c-Myc**, C-Master regulator of cell cycle entry, proliferative and metabolism, **CSCs**, Cancer stem cells, **CT**, The computed tomography, **DMSO**, Dimethyl sulfoxide, **DOPE**, Dioleoylphosphatidylethanolamine, **DOTAP**, 1,2-Dioleoyl-3-trimethylammonium propane, **DOX**, Doxorubicin, **DSPE**, 1,2-Distearoyl-sn-glycerol-3-phosphoethanolamine, **DTX**, Docetaxel, **EC**_**50**_, The half maximal effective concentration, ***E*-CAD**, E-cadherin, **EGFR**, Epidermal growth factor receptor, **EPR**, The enhanced permeability and retention, **ERK**, Extracellular regulated kinase, **EZH2**, Enhancer Of Zeste 2 Polycomb Repressive Complex 2 Subunit, **FAK**, Focal adhesion kinase, **FRα**, Folate receptor-α, **GalNAc**, *N*-acetylgalactosamine, **GEM**, Gemcitabine, **GnRH**, Gonadotropin-releasing hormone, **GSH**, Glutathione, **H1F1**, Hypoxia-inducible factor 1, **HRAS**, GTPase HRas enzyme, **IC**_**50**_, The half-maximal inhibitory concentration, **IL-17B**, Interleukin 17B, **ILK**, Integrin-linked kinase, **Kras**, Kirsten rat sarcoma GTPase enzyme, **LDL**, Low-density lipoprotein, **LHRH**, Luteinizing hormone-releasing hormone, **LHSSG2C**, Ditetradecyl 2-(4-(2-(2-(2-(2-(2,6-diaminohexanamido)-3-(1H-imidazole-4-yl) propanamido) ethyl) disulfanyl) ethylamino)-4-oxobutanamido) pentanedioate, **LRP**, Lung resistant protein, **MAPK**, Mitogen-activated protein kinase, **MDM**, Mixed dendrimer micelles, **MDR**, Multidrug-resistant, **miRNA**, Micro ribonucleic acid, **MRI**, Magnetic resonance images, **MSNRs**, Mesoporous silica nanorods, **MTDH**, Metadherin, **MTT**, 3-(4,5-Dimethylthiazol-2-yl)-2,5-diphenyltetrazolium bromide, **MVP**, Major vault protein, **NF-κB**, Nuclear factor-kappa light chain enhancer of activated B cells, **Notch-1**, Notch homolog 1, translocation-associated, **OEI**, Oligoethylenimine, **ORF**, Open reading frame, **OxaPt**, Oxaliplatin, **p27**^**Kip1**^, Cell cycle inhibitor, **PAH**, Poly (acrylhydrazine), **pAKT**, Phosphatidylinositol 3-kinase and Protein Kinase, **PAMAM**, Polyamidoamine, **PBS**, Phosphate Buffered Saline, **PDMAPMA**, Poly (3-dimethylaminopropyl methacrylamide), **pDNA**, Plasmid deoxyribonucleic acid, **PDX**, Patient-derived xenograft, **PEG**, Polyethylene glycol, **PEI**, Polyethyleneimine, **P-gp**, P-glycoprotein, **PI3-kinase**, Phosphatidylinositol 3′-kinase, **PLA**, Polylactic acid, **PLGA**, Poly (lactic-co-glycolic acid), **Polζ**, Translesion DNA polymerase, **PTEN**, Phosphatase and tensin homolog, **PTK-1**, Protein tyrosine kinase 1, **PTX**, Paclitaxel, **Q**, Combination efficacy, **R**, Resistance index, **Rac1**, Ras-related C3 botulinum toxin substrate 1, **RES**, Reticuloendothelial system, **REV**, Reversionless phenotype, **RGD**, The tripeptide arginine−glycine−aspartic sequence, **RISC**, RNA Induced Silencing Complex, **shRNA**, Short hairpin ribonucleic acid, **SIP-1**, Stress-induced protein 1, **siRNA**, Small interfering ribonucleic acid, **SLN**, Solid lipid nanoparticles, **SR-BI**, Scavenger receptor class B type I, **SSRTs**, Somatostatin receptors, **STAT-3**, Signal transducer and activator of transcription 3, **TGN**, Brain targeting peptide, **TIMP3**, Tissue inhibitor of metalloproteinase 3, **TLR4**, Toll-like receptor 4, **TLS**, Translesion synthesis, **TRAIL**, Tumor necrosis factor (TNF)-related apoptosis-inducing ligand, **USP9X**, Ubiquitin specific peptidase 9, X-linked, **VEGF**, Vascular endothelial growth factor, **ZEB**, Zinc finger E-box-binding homeobox 1 transcription factor

## Abstract

Chemoresistance and hence the consequent treatment failure is considerably challenging in clinical cancer therapeutics. The understanding of the genetic variations in chemoresistance acquisition encouraged the use of gene modulatory approaches to restore anti-cancer drug efficacy. Many smart nanoparticles are designed and optimized to mediate combinational therapy between nucleic acid and anti-cancer drugs. This review aims to define a rational design of such co-loaded nanocarriers with the aim of chemoresistance reversal at various cellular levels to improve the therapeutic outcome of anticancer treatment. Going through the principles of therapeutics loading, physicochemical characteristics tuning, and different nanocarrier modifications, also looking at combination effectiveness on chemosensitivity restoration. Up to now, these emerging nanocarriers are in development status but are expected to introduce outstanding outcomes.

## Graphical abstract

**Figure:**
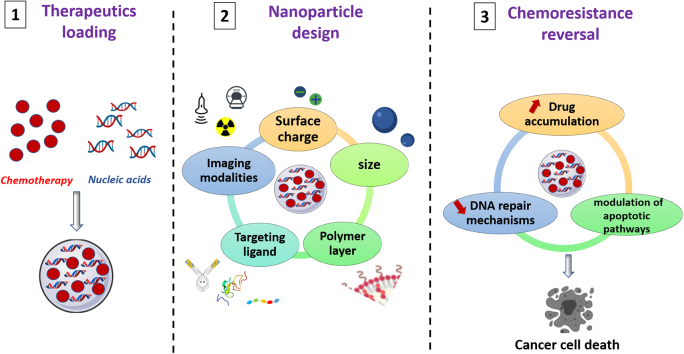

## 1 Introduction

According to the Global Cancer Incidence, Mortality and Prevalence (GLOBOCAN) database. Cancer is the second leading cause of death globally and was responsible for an estimated 9.9 million deaths in 2020 (Sung et al., 2021). Worldwide, about 1 in 6 deaths is caused by cancer (Bray et al., 2018). The available treatment options depend on surgery, radiation, classical chemotherapy, and recently developed targeted therapies such as tyrosine kinase inhibitors and immunotherapies. However, some severe dose-related toxicities are seen in most patients treated with anti-cancer drugs for an extended time, in addition to the intrinsic or acquired multidrug resistance (MDR) phenomenon. MDR decreases the drug accumulation inside the cell and increases the compensating mechanisms that alleviate the damage caused by these agents, eventually resulting in treatment failure with additional toxicities.

Chemoresistance can be induced at different cellular levels, either at efflux pump-mediated levels, involving different transmembrane and cellular carriers regulating drug concentration and availability to its cellular target, or at non-pump mediated levels through various modularity effects including DNA repair mechanism, apoptosis, and survival pathways to prevent cell death (Zheng, 2017). Up/down-regulation of the genes responsible for compensating mechanisms regulating cell death and DNA repair sensitize the cells more to the action of the chemotherapy, thus leading ideally to chemosensitivity restoration (Chen et al., 2018). Combining anti-cancer drugs with other therapeutic modalities with a distinct mechanism of action is evolving as a promising approach in treating the resistant type of cancer. Nucleic acids could be potentially included in many therapeutic strategies, as they target specifically and effectively anti-cancer drug-resistant genes. The combination should result in a synergistic enhanced therapeutic outcome and a restoration of the anti-cancer efficacy of the used drugs (Baguley, 2010; Zheng, 2017). RNA molecules have emerged as a new class of therapeutics that may permit the up/down-regulation of different cellular targets. Small RNA entities such as microRNA (miRNA), small interfering RNA (siRNA), and short hairpin RNA (shRNA) are introduced as modulators of the targeted gene expression and subsequent associated cellular effect. This modulation is achieved through the RNA interference (RNAi) mechanism, inducing either destruction of complementary post-transcriptional mRNA (siRNA) or suppressing its translation (miRNA) (Ichim et al., 2004; Kim and Rossi, 2008). Moreover, it has shown a valuable outcome in regulating the genes responsible for chemoresistance (Singh et al., 2008). DNA plasmids are circular, double-stranded DNA molecules independent of a cell's chromosomal DNA. Plasmids range in size from a few thousand base pairs to >100 kilobases (kb). To regulate the gene expression of interest, the exogenous DNA molecules are introduced into the host cells to regain the target gene function and hence promote its therapeutic action (Lodish et al., 2000).

Anti-cancer drugs targeting proliferating cells elicit their response through multiple mechanisms, often interfering with DNA biological functions and activating cell death pathways. In oncology practice, chemotherapy faces serious challenges, including inadequate tumor targeting, systemic side effects, and drug resistance, posing difficulties in anticancer drug development research. Similarly, nucleic acid delivery must overcome physiological barriers after systemic administration, including nonspecific interaction with plasma proteins, premature inactivation, and clearance from the circulation by the mononuclear phagocyte system (MPS), as well as its poor extravasation and tissue penetration into tumor cells. Moreover, overcoming the intracellular barriers is also needed to deliver nucleic acids successfully. A co-delivery system loading both chemotherapy and nucleic acids ensures that they undergo the same body distribution and kinetics, thus achieving the optimal benefits of the combination. For the simultaneous delivery of drugs and nucleic acids, while maintaining their physicochemical characteristics and biological functions, smart nanocarriers have been developed and optimized to achieve a successful combination for the designed aim of chemoresistance reversal. They include polymer-based nanoparticles (Devulapally et al., 2016; Pan et al., 2019), lipid-based nanoparticles (Oh et al., 2013; Reddy et al., 2016), and hybrid nanoparticles with an inorganic core (Liang et al., 2015; Ren et al., 2016; Zhang et al., 2017).

The purpose of this review is to address design considerations regarding therapeutics loading and nanoparticle modifications to mediate simultaneous administration of anti-cancer drugs and nucleic acids in the aim of chemoresistance reversal. This review is divided into three major parts. The first part describes the main aspects related to the principles of the therapeutics loading into the nanoparticles. The second part describes the required nanotherapeutics properties and the physicochemical characteristics for efficient delivery. The last part describes the selection criteria for nucleic acid targets, going through the therapeutic outcome of the combination delivery compared to individual therapies in the context of chemoresistance reversal.

## 2 Principles of therapeutics loading into nanoparticles

The combination of chemotherapy and nucleic acids using nanocarriers can be achieved either by delivering each modality separately or simultaneously. Some authors reported that the latter is more efficient due to convenience, synchronized pharmacokinetics, and delivery of defined amounts of both agents to a certain number of cells. In other words, it would be easier to create a rational cause-effect relation using multi-loaded carriers (Chen et al., 2010b; Li et al., 2013). Drug loading can occur at any time point during the formulation process. It can be made either during the nanoparticle generation or afterward by loading drugs in the core or at the surface of the nanoparticles. However, the suitable nanocarrier for combination therapy must have the ability to combine both types of therapeutic agents with acceptable physicochemical characteristics without interfering with each other and ensuring the drugs are released at the desired site of action. To avoid interactions between anti-cancer drugs and nucleic acids in rational nanoparticle design, therapeutics loading is done sequentially. In general, chemotherapy is launched first, followed by nucleic acid loading, as shown below. Nonetheless, some studies mentioned a co-loaded strategy in the same space, with specific preauction, but this is not the most common way to combine therapeutics in a co-delivery system.

### 2.1 Loading of chemotherapeutic drugs

**Fig. 1:**
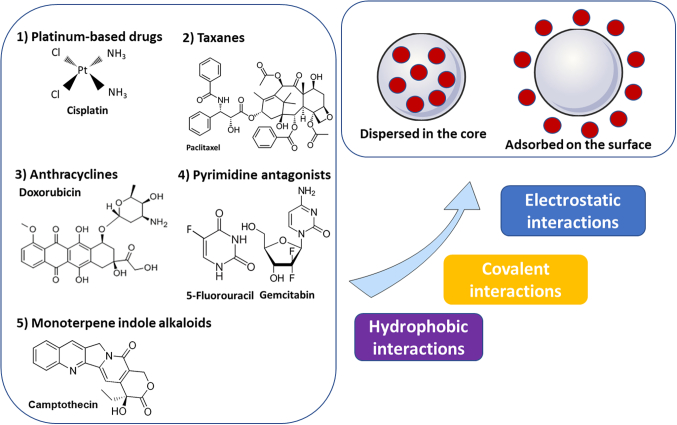
Anti-cancer drug loading into nanoparticles.

Chemotherapeutic drugs can be loaded into the nanoparticles according to their hydrophobicity-hydrophilicity nature, solubility profile, molecular weight, ability to chemically interact with a certain moiety, and susceptibility to further modification without the loss of their activity. Generally, drug loading varies and often occurs throughout the different fabrication processes with no exclusive method (Haag and Kratz, 2006). The following subsections present the loading of chemotherapeutic drugs into the nanoparticles according to the type of drug, with particular consideration for the dual loading with nucleic acids. A schematic overview is given in Fig. 1.

#### 2.1.1 Platinum-based alkylating agents

**Fig. 2:**
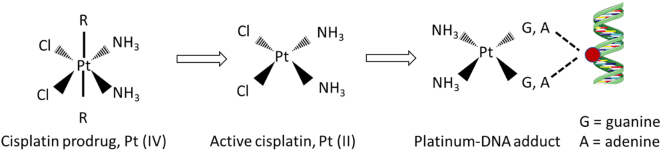
Cisplatin prodrug active form and interaction with nucleic acids.

The main consideration regarding this group is the chemical interaction between platinum-based drugs and nucleic acids, which seems to interfere with the silencing activity mediated through nucleic acids. In other words, their interaction leads to premature inactivation of the nucleic acids (Hedman et al., 2011; Polonyi et al., 2014). Considering this, delivery systems that use both nucleic acids and platinum-based drugs in the same cargo either physically separate the two by layers to inhibit their interaction or utilize an un-reactive platinum species. Once reaching the cell, the un-reactive prodrug is activated and can form DNA adducts with double-stranded DNA, as illustrated in Fig. 2. Loading of Cisplatin (CisPt) as a prodrug, like CisPt (IV) in the oxidation state to avoid platination with nucleic acids, was described by Yu et al. They developed iron nanoparticles covalently linked with CisPt (IV) with a loading capacity of 11.6% (*w*/w). In the intracellular environment, CisPt (IV) was reduced to form the active form CisPt (II), which interacts with its cellular target to elicit its anti-tumor effect (Yu et al., 2018). Similarly, Babu et al. described the incorporation of CisPt into the core of PLA-based nanoparticles. CisPt-containing nanoparticles were prepared by nanoprecipitation and achieved loading efficiencies up to 82%. To physically separate nucleic acids from CisPt, siRNA and plasmid DNA were electrostatically attached to the nanoparticle surface. Furthermore, this loading order resulted in a sequential release. Indeed, nucleic acids were released first, then CisPt was released in a controlled manner for up to 72 h under physiological conditions (Babu et al., 2014). Furthermore, Xiao et al. developed polymeric micelles to combine anti-Bcl-2 siRNA and Oxaliplatin (OxaPt (IV)), another member of platinum-based drugs. Inside the micelles, the siRNA was protected from platination mediated by platinum species by two mechanisms: i) physical separation in different layers between the two, as siRNA was electrostatically combined in the polymer corona and OxaPt (IV) was covalently linked inside the core; ii) utilization of an unreactive OxaPt (IV) analog, which can be reactivated inside the cells to form free OxaPt (II). The free OxaPt (II) can then interact with the nuclear DNA to induce the therapeutic effect. More precisely, they demonstrated that the combination does not only promote siRNA transcript stability but also induces a maximal suppression of mRNA levels, more than that ensured by either agent alone or when combined by another means (Xiao et al., 2017).

#### 2.1.2 Taxanes

Following the rule “like dissolve like,” paclitaxel (PTX), a well-known hydrophobic drug, can be incorporated into the nanoparticle's hydrophobic core. One interesting example of PTX loading into the nanoemulsion was achieved through solubilization in a lipid-based iodinated derivative, Lipiodol®. Using this method, the loading efficiency achieved was up to 98.5% (Oh et al., 2013). Another example of PTX loading within the hydrophobic core was reported in the work of Zhu et al. They used N-succinyl chitosan (NSC) conjugated with a reduction-sensitive linker cystamine (SS) and pH-sensitive linker urocanic acid (UA) to form NSC-SS-UA nanocarriers. NSC established the hydrophilic shell, while cystamine and urocanic acid constituted the hydrophobic core of the nanoparticles. PTX was loaded into the hydrophobic core to form PTX/NSC-SS-UA, with PTX loading up to 94.81% ± 1.37%. Then, low-density lipoprotein (LDL) conjugated siRNA was grafted on the surface of their nanoparticles through an amide bond (Zhu et al., 2017). PTX can also be encapsulated physically in self-assembled cationic micelles. Hu et al. utilized PTX modified with adamantine (Ada) *via* an amide link to obtain 2-amineadamantine-conjugated PTX (Ada-PTX) to construct the hydrophobic core of their supramolecular micelles. Adamantine served as a hydrophobic moiety, which can incorporate PTX for efficient drug loading. β-cyclodextrin-poly-ethylenimine (PEI-CyD) was used as a cationic shell that self-assembled with Ada-PTX to form the cationic micelles and adsorb shRNA on the surface (Hu et al., 2012).

Docetaxel (DTX), another member of the taxane group, can also be loaded into the nanoparticles following the same rule. Zheng group reported the incorporation of DTX into their polypeptide self-assembled micelles during the synthesis process by dissolving DTX into the *co*-polymers in the presence of dimethyl sulfoxide (DMSO). The drug was released at the cellular level with an enhanced therapeutic profile and less toxic events compared to the free drug (Zheng et al., 2013).

#### 2.1.3 Anthracyclines

**Fig. 3:**
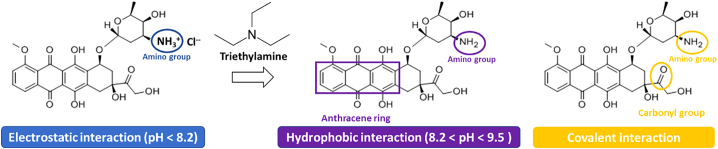
Types of interactions used to load Doxorubicin into nanoparticles.

Anthracyclines are a class of drugs considered among the most effective anti-cancer treatments. The most important anthracyclines are Doxorubicin (DOX), Daunorubicin, Epirubicin, and Idarubicin. We decide to highlight DOX as it serves as a model drug for this group, and it is widely used because it possesses various interaction sites and tunable characteristics according to pH. Moreover, DOX is a small molecule containing an anthracene ring that gives the DOX its hydrophobic nature and fluorescence properties, and it is functionalized with one amino group responsible for its positive charge at pH below 8.2 (Weiss, 1992), as shown in Fig. 3. DOX has a broad-spectrum therapeutic effect against different types of cancer, and it is excessively used in combination with nucleic acids. DOX was efficiently incorporated into different nanoparticles for dual nucleic acid/drug therapy.

Doxorubicin contains flat aromatic rings that intercalate into the DNA helix. This intercalation ability contributes to its loading ability into the delivery systems containing DNA molecules in their backbone (Eckel et al., 2003). Chen et al. described the use of DNA as a DOX carrier to mediate siRNA loading with DOX at the same time. They combined DOX with DNA duplex through physical intercalation into their liposome-polycation-DNA (LPD)-based nanoparticles and siRNA. The results confirmed that DOX loading through intercalation did not change the property of DOX or siRNA in the LPD nanoparticles. Moreover, the delivery of the encapsulated DOX was much more efficient than with free DOX (Chen et al., 2010a, Chen et al., 2010b). In the same manner, Yue et al. loaded DOX into DNA functionalized gold nanoparticles at the intercalating site (5’-GC-3′ or 5’-CG-3′) in the DNA duplex. DOX was released during the disassembly process of the nanocarrier (Yue et al., 2020). As DOX might interfere with DNA's therapeutic action, the DNA functionality must be considered when combined with DOX for gene modulatory purposes. Davoodi et al. loaded DOX in the core of their PEI-based polymeric nanoparticles, while the plasmid DNA was electrostatically assembled on the surface of the nanoparticles. The results showed that the presence of DOX did not influence the plasmid DNA integrity, functionality, or gene modulatory effect (Davoodi et al., 2016). Fig. 3 summarizes the types of interactions for DOX loading into the nanoparticle core or on their surface mentioned in this part.

The positively charged DOX molecule, due to its amine groups can also be electrostatically loaded into the nanoparticles (Fig. 3). Moreover, DOX can be presented in two forms: (i) as a monomer, which possesses an anti-cancer effect, and (ii) as a dimer with no efficacy by itself (Menozzi et al., 1984). All attempts to encapsulate DOX chose a way to avoid DOX dimerization in aqueous media. Alinejad et al. utilized polymeric conjugation to combine DOX *via* electrostatic interactions, which is considered the best way to avoid DOX self-association. DOX was loaded with an encapsulation rate of up to 75% into nanoparticles containing both anionic carboxymethyl dextran (CMD) and cationic chitosan nanoparticles. Two electrostatically stabilized complexes were mixed together (i) negatively charged CMD formed electrostatic interaction with positively charged DOX, and (ii) negatively charged siRNA interacting with positively charged chitosan. The loaded DOX and siRNA significantly induced cell apoptosis and confirmed the therapeutic action of the payload (Alinejad et al., 2016). Ren et al. described the successful electrostatic loading of DOX in their negatively charged hollow gold nanoparticles (HGNPs). HGNPs were porous, which facilitates DOX entry inside the holes. These pores enable the electrostatic interaction between the two, from outside and inside. This property made HGNP successfully loaded with a high payload of DOX 4.8-fold increase compared to solid gold nanoparticles with the same surface charge and weight. miRNA was bound electrostatically on the surface of DOX-loaded HGNP. This system released DOX 4 h after miRNA release in response to HGNP heating employing near-infra-red (NIR) light. This sequential drug release profile had a much better therapeutic outcome than synchronizing burst release of the therapeutics (Ren et al., 2016). Zhao's group used an easy coprecipitation method to encapsulate DOX in their alginate calcium carbonate hybrid nanoparticles. This loading was attributed to two factors: (i) calcium carbonate in the presence of alginate has many nanopores, which had the ability to contain the drugs regardless of the hydrophobicity or surface charge by capillary force, and (ii) alginate bears a negative charge which can easily electrostatically bind the positive DOX. The encapsulation efficiency was high, up to 84.2%, and the release of DOX was sustained for >48 h (Zhao et al., 2012).

**Fig. 4:**
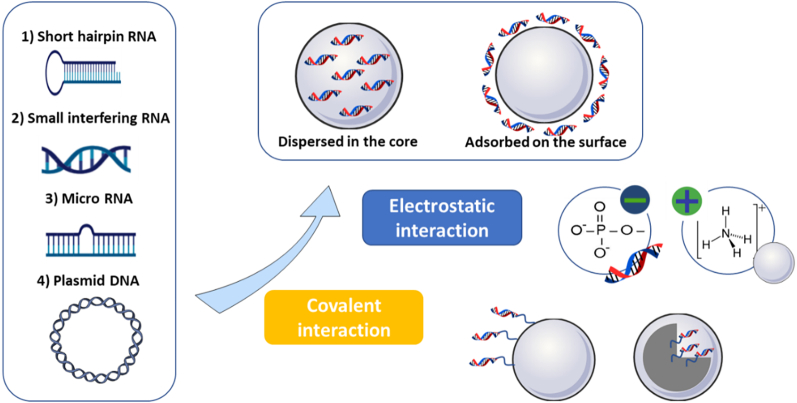
Nucleic acid loading into nanoparticles.

Also, the hydrophobic nature of DOX enables its loading in hydrophobic locations. Han et al. transformed DOX-HCL into hydrophobic DOX by addition of triethylamine (pH > 8.2) to be able to incorporate the molecular form of DOX into their polymer nanocarriers (Fig. 4). The nanocarrier was composed of polyamidoamine (PAMAM) dendrimers decorated with Hyaluronic acid (HA). DOX was embedded in the interior hydrophobic core of the nanocarrier (1:10 weight ratio, DOX to polymer), and siRNA was electrostatically bound on the surface. In phosphate-buffered saline (PBS) at pH ≈ 7.4, DOX was released from the core of the nanoparticles in a controlled manner for >96 h (Han et al., 2012). Kim et al. also transformed DOX-HCl into hydrophobic DOX before loading it into polymersomes with a hydrophobic shell based on PEG-Poly-lactic acid (PLA). The DOX loading efficiency was 32.2%. These polymersomes had the ability to combine hydrophobic DOX in their shell and hydrophilic siRNA in their core. Once in endosomes, the release of DOX was favored due to acid hydrolysis of PLA polymer. They also reported a faster release of DOX than siRNA, which was attributed to the DOX presence in the shell, while siRNA was located inside the core of the nanoparticles (Kim et al., 2013).

Another type of DOX loading involves covalent interactions between DOX and the nanoparticles (Fig. 3). Suo et al. linked the carbonyl group of DOX with the hydrazine group of poly-acrylhydrazine presented in their PEGylated triblock *co*-polymer nanoparticles. This covalent linkage resulted in high loading efficiencies. The pH-responsive hydrazone bond was cleaved in acidic media to release DOX. Thus, DOX was found to be accumulated in cytoplasmic and nuclear regions to exert its anti-cancer effect (Suo et al., 2017). Following the same concept, Xu et al. developed PEI-based nanoparticles with pH-sensitive linker cis-aconitic anhydride (CA). CA interacted with the DOX amino group through cis-aconityl linkage. The DOX content in PEI-CA-DOX nanoparticles was ≈10.2%. This linkage was cleaved in acidic pH and led to DOX release over 72 h (Xu et al., 2015).

#### 2.1.4 Anti-metabolites - pyrimidine antagonists

5-fluorouracil (5-FU) is widely used as an anti-tumor drug, but only a few studies reported the encapsulation of 5-FU. The main way to encapsulate 5-FU was achieved through electrostatic interaction as 5-FU is a weak acid and negatively charged. Chen's group reported the encapsulation of 5-FU into the chitosan nanoparticles during the synthesis process using the ionic gelation method. In the presence of the polyanion tripolyphosphate and acidic media, 5-FU is encapsulated efficiently up to 44.28 ± 1.69% (*w*/w) (Chen et al., 2017a). Li et al. used the same approach to encapsulate anionic 5-FU into double-layered hydroxide nanoparticles with loading capacities of up to 22.6% (w/w) (Li et al., 2014).

Gemcitabine (GEM) is a nucleoside analog used to treat various types of cancers. It can be incorporated into the nanoparticles with the simultaneous presence of nucleic acids. Most studies reported the incorporation of GEM during the synthesis process of the nanoparticles. Of these studies is the one by Zhang et al., where they loaded Gemcitabine monophosphate by electrostatic interaction into the core of calcium phosphate-based nanoparticles using the water-in-oil micro-emulsions method. They attained a loading efficiency of up to 75%. The cells readily uptaken these nanoparticles loaded with GEM, and Gemcitabine monophosphate underwent phosphorylation by cellular nucleoside kinase to exert its therapeutic action (Zhang et al., 2013).

#### 2.1.5 Monoterpene indole alkaloids

Camptothecin (CPT) is a monoterpene indole alkaloid considered a promising anticancer drug that exerts its therapeutic efficacy by inhibiting topoisomerase-I. Camptothecin has a low water solubility and poor biostability due to the conversion of the active lactone ring to the inactive carboxylate form in plasma. Babaei et al. designed PEGylated rod-shaped mesoporous silica nanorods (MSN) that served as a biocompatible nanocarrier to simultaneously deliver Camptothecin and survivin shRNA-expressing plasmid (iSur-DNA) to colon adenocarcinoma cell lines. Camptothecin was loaded into the MSN with an encapsulation efficiency of 32% due to the inherent properties of MSN. The drug was adsorbed into the large pores provided by the MSN structure. Moreover, positively charged NH_2_-MSN can be electrostatically interacted with reactive sites on Camptothecin molecules mainly (oxygen and nitrogen) (Babaei et al., 2020).

### 2.2 Loading of nucleic acids

For instance, nucleic acids can either be loaded into nanoparticles through electrostatic interactions or after chemical modification *via* covalent linkage to the nanoparticles, as illustrated in Fig. 4.

#### 2.2.1 Loading through electrostatic interactions

Due to the presence of phosphate groups, nucleic acids bear numerous negative charges on their surface, enabling them to interact with positively charged components of the nanoparticles (Fig. 4). Several positively charged components (*i.e., cationic polymers, cationic lipid ligands, …etc.*) were specially designed or selected for this.

It is important to mention that all nucleic acids can be electrostatically loaded into the nanoparticles either on the surface or in the core using different cationic materials, as illustrated in Fig. 4. Oh et al. reported that siRNA was adsorbed on the positive surface of their nanoemulsion, which was composed of cholesterol, linear polyethyleneimine (PEI), and a phospholipid-polymer conjugate composed of 1,2-Distearoyl-sn-glycerol-3-phosphoethanolamine (DSPE) and polyethylene glycol (PEG). PEI constituted the main cationic element in their system, promoting the electrostatic interaction with siRNA (Oh et al., 2013). Babu et al. designed nanoparticles based on polylactic acid (PLA). The PLA core was then coated with the cationic polymer chitosan for efficient loading of both siRNA and plasmid DNA at the surface of their nanoparticles (Babu et al., 2014). Furthermore, Chen et al. described the electrostatic loading of an anti-survivin siRNA in their liposomes-based nanosystem through the interaction between anionic siRNA and a redox-sensitive cationic lipid Ditetradecyl 2-(4-(2-(2-(2-(2-(2,6-diaminohexanamido)-3-(1H-imidazole-4-yl) propanamido) ethyl) disulfanyl) ethylamino)-4-oxobutanamido) pentanedioate (LHSSG2C_14_). In the reductive cytosol conditions, siRNA was released due to the reduction of the disulfide bonds of this cationic lipid (Chen et al., 2017b). In contrast to the previous examples, Zhang et al. reported the loading of siRNA into the core of lipid calcium phosphate nanoparticles. The nucleic acid loading was mediated by electrostatic interaction between the phosphate group of the nucleic acid and the calcium ion constituting the nanoparticle's core (Zhang et al., 2013). Devulapally et al. used electrostatic interactions between miRNA-21 and the counterion spermidine to enhance the solubility of highly anionic miRNA-21 in Poly (Lactic-glycolic acid) (PLGA). Their PEGylated- PLGA-based nanoparticles were obtained by the water-in-oil-in-water (w/o/w) double emulsion method and encapsulated the miRNA in their hydrophobic PLGA core (Devulapally et al., 2016).

#### 2.2.2 Loading after chemical modifications of nucleic acids

As nucleic acids are hydrophilic in nature, their chemical interactions with hydrophobic elements could be facilitated through chemical conjugation with certain moieties and linkers. For example, Zhu's group reported modification of siRNA by chemical conjugation with cholesterol, which was then successfully incorporated into a low-density lipoprotein layer on the surface of their polymeric micelles (Zhu et al., 2017). Other approaches reported a chemical modification that was achieved using a redox-pH sensitive linker with either modified or unmodified nucleic acids. He et al. used thiol conjugated siRNA to form phospholipid DSPE-siRNA conjugates through disulfide bonds. These phospholipid DSPE-siRNA conjugates can later be included in the lipid shell layer of nanoscale coordination polymers (NCP). NCP are self-assembled from metal ions (Zn) and organic bridging ligands (bisphosphonate ligands). At the cellular level, the disulfide bonds between DSPE and siRNA were cleaved, releasing siRNA (He et al., 2016). Salzano et al. described the reversible conjugation of unmodified siRNA with phospholipid (phosphatidylethanolamine, PE) *via* a disulfide linkage to form siRNA-S-S-PE, which further interacts with PEG in the developed polymeric micelles. In this construction, siRNA was released in the reductant conditions inside the cells (Salzano et al., 2015).

## 3 Required nanotherapeutics properties for an efficient delivery

Nanocarriers have to be designed in a manner that could promote stability and structural integrity for further enhanced effectiveness. Hereafter, we go through various combination mediated nanoparticle properties, physicochemical characteristics such as size and charge, and surface modifications like the grafting of a polymer layer and/or the presence of active targeting ligands. Moreover, nanotherapeutics have to be designed in a way to circumvent several steps, including serum stability, cell endocytosis, endo-lysosomal escape, and efficient release of the drug/nucleic acid entities. It is nice to mention that all these modifications are chosen in such a manner to work in harmony together to provide the interesting fea tures of such a co-loaded modality. Although many features can be integrated to alleviate nanoparticle properties for a mono delivery, our primary focus was related to the co-delivery aspect.

### 3.1 Moderate size

The size is considered one of the most critical parameters in nanoparticle design for drug delivery. A suitable nanocarrier for therapeutic purposes should form nanoparticles within a nanometer scale to facilitate the nanoparticle's cellular binding and endocytosis and subsequently improve its transfection efficiency. It should also avoid immune system recognition, renal clearance, and size-dependent cytotoxicity (Bhatia, 2016). The nanoparticles uptake and accumulation used in cancer therapeutics are in part dependent on what is called the enhanced permeability and retention (EPR) effect. It is defined by an aberrant vascular architecture, that promotes extravasation within tumor tissues as a result of increased production of vascular permeability factors and the lack of lymphatic drainage. These characteristics of tumor neoangiogenesis, in combination with the properties of the nanoparticles, especially a size below 200 nm, favor the nanoparticle accumulation in the tumor microenvironment (Azzopardi et al., 2013; Golombek et al., 2018a; Greish, 2010).

Table 1 illustrates the size evolution upon therapeutics loading and the addition of different ligands. It is logical to notice an increase in nanoparticle size from their basic structure to their final form due to anticancer drugs, nucleic acid drugs loading, and/or surface modification with various ligands. However, the degree of size increment is dependent on many considerations. The use of nanotechnology precisely provides controllable, modifiable nanoparticles in terms of their size, surface functionality, and, therefore, their therapeutic response. In the literature, the nanosystems that combine nucleic acids and cytotoxic drugs simultaneously reported an average size between 150 and 200 nm, which is ideal for an efficient therapeutic outcome (Han et al., 2014; Reddy et al., 2016). This reasonable size allows to overcome the physiological barriers that govern the NP's uptake, stability, and clearance. However, conserving this size range is difficult and sometimes became a big hurdle in the progress of the finely engineered nanocarriers for combinational delivery, because of the lower drug-loading capacity per particle and the difficulty to maintain a homogeneous size distribution.

**Table 1:** Size and zeta potential evolution in response to the addition of drug, nucleic acids, and different modifications.

Basic Nanoparticle			Types of components added			Component addition order	Final NP		Study type	References
NP type	Size (nm)	Zeta value (mV)	Drug	Nucleic Acid type	Modification		Size (nm)	Zeta value (mV)		
**1. Polymer-based nanoparticles**										
PLGA based NP	95.3 ± 7.3	−35.7 ± 7.8	PTX	siRNA	PEI	PEI-PTX-siRNA	295.3 ± 14.6	+40.8 ± 6.6	*In-vitro*	Su et al., 2012
PLGA based NP	229.2 ± 5.0	−5.28 ± 0.40	PTX	siRNA	Hyaluronic acid	PTX-siRNA-Hyaluronic acid	232.9 ± 06.9	−6.99 ± 0.42	*In-vitro/* *In-vivo*	Byeon et al., 2018
Dendrimer Micelles	41.06 ± 0.61	+20.40 ± 3.39	DOX	siRNA	PEG+DOPE	PEG+DOPE-DOX-siRNA	175.8 ± 1.04	+4.55 ± 0.25	*In-vitro*	Pan et al., 2019
PLA based NP	–	–	CisPt	siRNA, pDNA	Chitosan layer	CisPt-Chitosan-siRNA-pDNA	≈350	+5	*In-vitro*	Babu et al., 2014

**2. Lipid-based nanoparticles**										
Nanoemulsion	132 ± 0.6	+59.7 ± 0.7	PTX	siRNA	–	PTX-siRNA	178.8 ± 1.5	+43.0 ± 0.3	*In-vitro/* *In-vivo*	Oh et al., 2013
Solid lipid NP	82.1 ± 2.9	+41.5 ± 2.6	DOX	pDNA	Transferrin	DOX-pDNA-Transferrin	286.5 ± 3.9	+19.1 ± 1.8	*In-vitro/* *In-vivo*	Han et al., 2014
Liposomes	120.0 ± 10.0	+40.5 ± 1.2	PTX	siRNA	–	PTX-siRNA	136.0 ± 3.0	+34.5 ± 1.3	*In-vitro/* *In-vivo*	Reddy et al., 2016
Liposomes	76.7 ± 2.4	+25.5 ± 1.8	PTX	siRNA	–	PTX-siRNA	156.4 ± 2.1	+15.2 ± 1.4	*In-vitro/* *In-vivo*	Chen et al., 2017b

**3. Hybrid nanoparticles with inorganic core**										
Iron-based NP	≈29.7	–	CisPt	siRNA	PEI, PEG-LHRH targeting peptide	CisPt-PEI, PEG-LHRH targeting peptide-siRNA	≈423.8	+22.9 ± 0.5	*In-vitro/* *In-vivo*	Yu et al., 2018
Alginate/CaCO_3_ Hybrid NP	–	–	DOX	pDNA	–	DOX-pDNA	145.0 ± 7.8	−13.8 ± 1.8	*In-vitro*	Zhao et al., 2012

**CisPt**: Cisplatin, **DOPE:** Dioleoylphosphatidylethanolamine, **DOX:** Doxorubicin, **LHRH**: Luteinizing hormone-releasing hormone, **pDNA:** Plasmid deoxyribonucleic acid, **PEG:** Polyethylene glycol**, PEI:** Polyethyleneimine, **PLA:** Polylactic acid, **PLGA:** Poly (lactic-*co*-glycolic acid), **NP:** Nanoparticle, **PTX:** Paclitaxel, **siRNA:** small interfering ribonucleic acid.

There were also reports of an exceptional size value, where the size is relatively small. For example, Salzano et al. used polymeric micelles with a size around 25.0 ± 3.6 nm that combined survivin siRNA and Paclitaxel (Salzano et al., 2015). Also, Li et al. developed quantum dots (QD) nanoparticles with a core diameter of 5 nm that can co-deliver Bcl-2 siRNA, and different anticancer drugs include Paclitaxel, Carboplatin, and Doxorubicin. They proved that their nanoparticles form different stable complexes dependent on the molecular size of the loaded drug for *in vitro* application. These nanoparticles can be used for both therapeutic and theranostic purposes due to the QD's strong fluorescence properties allowing real-time imaging of the drug delivery and release (Li et al., 2016). Nanoparticles with a big size of ∼550 nm were reported by Wu et al. using silica-based nanoparticles loading P-gp siRNA with Doxorubicin in this large pore-sized hollow mesoporous organosilica, and the siRNA loading is up to 200 μg/mg. This design allows therapeutics high loading capacity and adequate siRNA protection from enzyme degradation compared to other mesoporous materials-based gene-delivery nanocarriers (Wu et al., 2016). Anticancer drugs are usually small molecules with variable sizes, so the expectance of the size change depends on the drug and the concentration of the loaded drug according to its subsequent application for *in vitro* or *in vivo* studies. Nucleic acids also had a small size with intra-size variation between DNA plasmid, miRNAs, shRNA, and siRNA.

The size of siRNA, for example, is about 7 nm. The increase in nanoparticle size is also dependent on the siRNA loading method. When the siRNAs are loaded through chemical conjugation, siRNA can form a double- or a multilayer, which provides additional size increment. On the other hand, when nucleic acids are loaded through electrostatic interactions with cationic polymers, the NPs size increases after the polymer grafting and decreases after the final nucleic acid drugs loading, as explained by Gao et al. They synthesized polyplexes to co-deliver siMDR and Doxorubicin. With the increase of the N/P ratio from 1 to 8, particle size decreased from 658 ± 22.74 nm to 116 ± 13.8 nm because of the increase in the electrostatic attraction forces (Gao et al., 2019). Another consideration is the method used for size measurement. Determining the nanoparticle size is generally made by Dynamic Light Scattering (DLS), where the nanoparticles are in dispersion. The DLS gives hydrodynamic sizes, which estimate the size of the nanoparticle core plus the liquid layer around the particle. The other widely used method is Transmission Emission Microscopy (TEM) which determines the nanoparticle's core size in a dry state. That is why the two methods report a difference in size. The nanoparticles used for combination therapy usually present a core-shell structure to carry the anticancer drug in the core and the nucleic acids in the shell or *vice versa*; that is why the use of DLS is more favorable than TEM. For example, Messaoudi et al. loaded nucleic acid *via* electrostatic interaction after surface modification with hydrophilic polymers. Given the small size of siRNA (∼7 nm), the considerable increase in the nanoparticle hydrodynamic diameter is related to siRNA and water molecules associated with siRNA that give a hydration layer detected by DLS (Messaoudi et al., 2014).

### 3.2 Positive surface charge

Zeta potential value is an indirect measure of the surface charge of the nanoparticles, controlling many vital aspects regarding formulation stability and biological fate of the nanoparticles, including the colloidal stability of the nanoparticles in dispersion by charge repulsion forces, the nanoparticle's stealthiness against the reticuloendothelial system (RES) opsonization, and nanoparticles charge mediated cellular uptake and cytotoxicity (Shah et al., 2015). The nanoparticles get their charge depending on the nature of the material used in the synthesis process, their surface functionalization, and therapeutics loading, as presented in Table 1. The reported zeta potential values in the combinational model generally were positive. This positive surface charge differs from the surface charge of the nanoparticles designed for example of anti-cancer mono delivery where the surface charge is neutral in most of the cases. This surface charge shift can be explained by the therapeutic loading method, the sequence and location of the loaded therapeutics in the nanoparticles, whether the anti-cancer drug was loaded first or later, and the grafting of cationic elements that contribute to electrostatic loading of nucleic acids. The presence of positive charge anti-cancer drugs at the interface or surface of the nanoparticles can also contribute to the surface charge positivity, and this positive charge is proportional to the loaded anti-cancer drug concentration; when the drug concentration increased the positive surface charge increases. When the surface of the nanoparticles is modified with a polymer layer, for example, to condense nucleic acids, the zeta potential is gradually increased, whereas the electrostatic loading of nucleic acids will reduce the zeta potential. For example, Su et al. used poly (lactic-*co*-glycolic acid) based nanoparticles with a zeta potential of ≈ − 35.8 ± 7.8 mV. After the functionalization with PEI, the obtained nanoparticles' zeta potential values were increased to +53.6 ± 9.8 mV. After electrostatic loading of signal transducer and activator of transcription-3 (STAT-3) siRNA, the zeta potential decreased to +46.2 ± 7.1 mV. The reduction of the zeta potential value was attributed to the successful electrostatic complexation between the nucleic acid phosphate groups and the amino groups of the polymers presented at the nanoparticle surface (Su et al., 2012).

To reduce charge-mediated toxicity in the polymer-based nanoparticles. The loading of nucleic acids should be optimized regarding the N/P ratios. In return, slightly positive charged nanoparticles can facilitate the interaction with the negatively charged cell membranes promoting the uptake of the nanoparticles, and can be of interest to target specific organs such as the lung. In this context, Yu et al. developed an iron-based nanocarrier functionalized with a PEI layer to adsorb siRNA at an N/P ratio of 30:1. This adapted ratio led to the avoidance of charge-mediated cytotoxicity and provided a slightly positive charge of about +22 mV that was favorable for cellular uptake (Yu et al., 2018). Another interesting example of the charge-mediated uptake is presented by Shen et al. They developed polypeptide nanoparticles with a positive charge ≈ +30 mV, containing PEI/DNA plasmid expressing shRNA anti-survivin. These nanoparticles showed an *in vivo* lung tumor penetration due to three factors: (i) the EPR effect, (ii) the cyclic RGD (arginine glycine aspartic acid) active tripeptide to target integrin αvβ3 and neoropilin-1 protein presented on lung endothelial cells, and (iii) the positively charged nonspecific interaction of PEI/DNA complexes with plasma proteins such as immunoglobulin M (IgM), fibrinogen, and fibronectin. These latter aggregates were smoothly uptaken by lung tissues. Collectively this leads to additional selective delivery to lung cells (Shen et al., 2014; Zhong et al., 2013).

### 3.3 A stealth polymer layer

The immune system rapidly clears bare nanoparticles and consequently cannot exert their therapeutic action. Thus, the use of biocompatible shielding materials to mask the surface of the nanoparticles is of great importance. One of the most used materials to mediate this characteristic is polyethylene glycol (PEG). PEGylation describes the modification of the nanoparticles by covalent conjugation with PEG, which is a hydrophilic, non-toxic, and non-immunogenic polymer (Abuchowski et al., 1977a; Abuchowski et al., 1977b; Mishra et al., 2016). PEGylation changes the physicochemical properties of the nanoparticles and reduces the absolute zeta potential value due to the masking of the nanoparticle surface charges. As illustrated in the review by Hamidi et al., PEGylation also alters the pharmacokinetic properties of the nanoparticles. The changes occurred in overall circulation life-span, tissue distribution pattern, and elimination pathway compared to the encapsulated therapeutics alone or non-PEGylated nanoparticles (Hamidi et al., 2006). Generally, PEGylation decreases immunogenicity and increases nanoparticle stability and circulation time (Veronese and Mero, 2008). Nevertheless, PEGylation is also associated with the problem of controlling cellular uptake and endosomal escape owing to steric hindrance, which is called the “PEG dilemma” (Hatakeyama et al., 2013).

Cleavable PEG moieties can be used to circumvent this issue (Hatakeyama and Harashima, 2016). Jin et al. developed stable nanoparticles based on PLA with layer-by-layer delivery. These nanoparticles utilized PEG with poly (L-aspartic acid sodium salt) (PEG-PAsp), where the aspartic acid moiety is negatively charged at pH 7.4. This anionic polymer is electrostatically loaded onto the surface of cationic PEI-PLA NP that had been preloaded with PTX and siRNA. At pH below 6.0, for example, at the acidic pH of endosomes, the PEG-PAsp becomes neutral and is detached from the surface of the nanoparticles (Jin et al., 2018).

Following the same concept, Suo et al. conjugated PEG moieties with the poly (3-dimethylaminopropylmethacrylamide) PDMAPMA polymer using poly (acryl hydrazine) to form a redox-sensitive disulfide linkage. In the intracellular compartment, the disulfide bond is cleaved in response to glutathione (GSH) presence. This cleavage led to PEG detachment from the nanocarrier corona letting the PDMAPMA polymer initiate its endosomal escape through protonation and release the payloads to exert their desired therapeutic outcome (Suo et al., 2017).

Even if PEG is still considered the gold standard, it is not free from inconvenience. Indeed, some rare allergic responses to PEG can cause anaphylactic reactions, and the presence of PEG can induce the production of anti-PEG IgM that may accelerate blood clearance of the pegylated moieties. Alternatives to PEG are currently in development with the use of polysarcosine (pSAR), polyoxazoline (POx), and Poly (Zwitterions) (Hoang Thi et al., 2020).

### 3.4 Functionalization with active targeting ligands

The design of targeted nanoparticles that can deliver therapeutics at a predicted controlled rate directly to tumor cells may provide better efficacy and lower off-target toxicity, especially in the context of co-delivered therapeutics. In Table 2, we illustrate different targeting ligands that can selectively accumulate nanoparticles into tumor cells through either receptor-mediated or non-receptor-mediated endocytic pathways by the designed NPs for simultaneous delivery. This guided uptake is attributed to the fact that tumor cells overexpress specific types of receptors and cellular motifs more than normal cells (Yoo et al., 2019). All the listed modified nanoparticles increased the drug accumulation inside the tumor cells compared to non-targeted nanoparticles or free drugs and decreased chemotherapy-associated toxicity and unwanted drug accumulation. Therefore, they are considered a promising approach for tailored medicine in cancer therapeutics. The issue of targeting should be highly appreciated in co-loaded modality design, as the drugs and nucleic acids should be delivered in a certain amount to their site of action to exert their synergistic efficacy.

**Table 2:** Various active cell-targeting ligands utilized in nanoparticle-mediated combinational therapy.

Targeting ligand	Targeting receptor/cell motif	Cancer cell lines	Cancer type	References
**Proteins based ligands**				
Low-density lipoprotein	LDL receptors	MCF-7	Breast cancer	Zhu et al., 2017
Apolipoprotein A-I	SR-BI	MCF-7	Breast cancer	Wang et al., 2017
Transferrin glycoprotein	Transferrin receptor	A549	Lung cancer	Han et al., 2014

**Peptides based ligands**				
Asparagine-Glycine- Arginine peptide	CD13 receptor	HT-1080	Fibrosarcoma	Chen et al., 2010a
Luteinizing hormone-releasing hormone targeting polypeptides	GnRH receptors	A2780	Ovarian cancer	Yu et al., 2018
		A549, H-1975, PC-9, and PC-9GR	Lung cancer	Majumder and Minko, 2021
RGD acid	Integrin adhesion molecules αvβ3 and neoropilin-1 protein	A549	Lung cancer	Shen et al., 2014
		U87 MG	Glioblastoma	Yang et al., 2020
Angiopep-2 oligopeptide	LRP1	U87 MG	Glioblastoma	Sun et al., 2011
Vapreotide	SSRTs	MCF-7	Breast cancer	Feng et al., 2014
HAIYPRH T7 peptide	Transferrin receptor	U87 MG	Glioblastoma	Liu et al., 2012
Brain targeting peptide (TGN)	Cerebral vasculature	U87 MG	Glioblastoma	Yang et al., 2020
AS1411 DNA aptamer	Nucleolin protein	C26	Colon carcinoma	Babaei et al., 2020

**Small molecules ligands**				
Galactosylated ceramide	ASGPR	Huh7	Liver cancer	Oh et al., 2016
Folate	FRα	MCF-7	Breast cancer	Suo et al., 2017
Kojic acid	Melanocytes tyrosinase	B16F10	Melanoma	Reddy et al., 2016
Hyaluronic acid	CD44 receptor	MCF-7	Breast cancer	Han et al., 2012
Anisamide	Sigma receptor	H460, A549	Lung cancer	Zhang et al., 2013
Biotin	Biotin receptor	HeLa	Cervical carcinoma	Liang et al., 2015

**ASGPR:** Asialoglycoprotein receptor, **CD:** Cluster of differentiation, **FR:** Folate receptor, **GnRH:** Gonadotropin-releasing hormone, **LDL:** Low-density lipoprotein, **LRP1:** Low-density lipoprotein receptor-related protein, **RGD:** arginine−glycine−aspartic acid, **SR-BI:** Scavenger receptor class B type I, **SSRTs:** Somatostatin receptors, **TGN:** Brain targeting peptide.

For the receptor-mediated cellular uptake, Oh et al. developed liposomes linked to galactosylated ceramide for combined delivery of siRNA and DOX. These galactosylated liposomes were selectively accumulated in the asialoglycoprotein receptor (ASGPR) positive hepatocellular cell line Huh7 and showed less accumulation in ASGPR negative lung cancer cell line A549. Furthermore, they reported that DOX accumulated 4.8 folds higher in hepatocellular tumor tissues compared to free DOX and 2.3 times compared to non-galactosylated liposomes after *in vivo* administration. Besides, they observed that DOX accumulated less in cardiomyocytes, and siRNA was transfected efficiently into the cells. In sum, they concluded the overall enhancement of the therapeutic effect of the targeted liposomes that delivered the combination (Oh et al., 2016).

Furthermore, the ligand can provide additional features to the employed nanoparticles like biocompatibility, stability, and drug loading ability. For example, Zhu et al. reported the grafting of LDL on the surface of their polymeric micelles for specific LDL receptor targeting. Not only does this approach increase the cellular uptake of the nanoparticles through additional receptor-mediated endocytosis, but also, LDL renders the nanoparticles more stable and biocompatible. They also stated that LDL serves as a gene carrier and favors the dissolution of cholesterol conjugated siRNA into the hydrophobic portion of their micelles, as mentioned earlier (Zhu et al., 2017). Other studies reported using hyaluronic acid (HA), a biocompatible moiety. HA-decorated nanoparticles have a longer circulation time and less opsonization by the immune system compared to non-decorated nanoparticles. Also, HA provided conformational stability and enhanced binding selectivity for CD44 receptors (Han et al., 2012; Byeon et al., 2018).

Additionally, targeting ligands can reduce the nanoparticle's interaction with serum proteins, which is a crucial factor for co-delivery. Sun's research group confirmed that the conjugation of Angiopep-2 ligand on their cationic liposomes' surface facilitated the nanoparticle uptake into the glial cells. Moreover, the Angiopep-2 conjugation reduced the interactions with serum proteins, contributing to their liposomes' overall stability (Sun et al., 2011).

### 3.5 Ability to escape endo/lysosomes

Most nanoparticle delivery systems are internalized into cells through the endocytosis pathway (Canton and Battaglia, 2012). Uptake through endocytosis involves internalization into an endocytic vesicle, fusion into the early endosomal space, maturation into a late endosome, and accumulation in the lysosome. Generally, anti-cancer drugs passively diffused into the cells, while nucleic acids need to be vectorized in nanocarriers to be able to penetrate through the cell membrane *via* endocytosis. Hereafter, the co-loaded approach with the two therapeutics in one nanovector will need a further step of escaping the endosomal compartment to the cytoplasm where the loaded drugs are released to exert their therapeutic value. This step aspires to the design of nanocarriers in a way to promote anti-cancer/nucleic acid endosomal escape. More details concerning nucleic acid endosomal escape were better highlighted and investigated by Mendes et al. (Mendes et al., 2022).

#### 3.5.1 With the help of polymers possessing high proton buffering capacities

During the development of the endosome, the pH decreases from 7.4 to pH 6.5 in the early endosome, to pH 6.0 in the late endosome, and pH 5.0 in the lysosome (Iversen et al., 2011). Interestingly, endosome acidification plays a vital role in the “proton sponge” hypothesis mechanism of polyplex endosomal escape. This hypothesis explains that the buffering capacity of polyamine carriers leads to osmotic rupture of the endosomal membrane and hence the release of its loaded content into the cytosol (Boussif et al., 1995). The most used polyamine is PEI, but alternatives such as oligoethylenimine (OEI), Poly (3-dimethylaminopropyl methacrylamide) (PDMAPMA), and polyamidoamine (PAMAM) were also used.

PEI is widely used as a non-viral carrier for nucleic acids, promoting its endosomal escape due to its high buffering capacity, resulting in high transfection efficiency. At the same time, PEI positive charges can facilitate cellular uptake by electrostatic interaction with the negatively charged cell membrane. Nevertheless, it contributes also to the charge of the nanoparticles mediated cytotoxicity. This toxicity is mainly dependent on the molecular weight of the used PEI. For example, 1.8 kDa PEI showed less cytotoxicity and transfection efficiency than 25 kDa PEI (Kim et al., 2005). However, the total concentration of PEI is also important. Su et al. reported using high molecular weight PEI (25 kDa) in their PLGA-based nanoparticles. In this case, they did not induce significant toxicity due to the low total concentration of PEI in the final formulation (about 6 ng/μg PLGA) (Su et al., 2012). It was reported that PEI-induced toxicity begins from 10 μg/μL of PEI for 25 kDa PEI (Gwak and Kim, 2008). PEI with different molecular weights and degrees of branching with/without conjugation with alkyl chains, lipids, PEG…*etc.* was also stated to achieve a high transfection efficacy with a reduction of cytotoxicity. To avoid the limitation of using PEIs, Oligoethylenimine (OEI), *i.e.*, PEIs with a molecular weight <2 kDa, was used by Gao et al. (Gao et al., 2019).

PDMAPMA has previously demonstrated the proton buffering capacity similar to PEI due to its cationic dimethylaminopropyl groups, but with minimal cellular toxicity (Kirkland-York et al., 2010). Qian et al. developed biodegradable polymeric micelles using PLA-PDMAPMA with amphiphilic star-branched copolymers to form three different micelles architectures: PLA-PDMAEMA_3_, (PLA-PDMAEMA_3_)_2,_ and (PLA-PDMAEMA_3_)_3_. PLA polymer was used for biodegradability, while PDMAPMA was used for effective endosomal escape due to its buffering ability to induce the “proton sponge effect.” All three star-branched copolymers exhibited low cytotoxicity compared to PEI. The cytotoxicity of the micelles is dose-dependent cytotoxicity, and it increased proportionally to the number of arms of star branched copolymer used. The (PLA-PDMAEMA_3_)_3_ optimized micelles had much lower cytotoxicity and 2.5 times higher transfection efficiency compared to PEI (25 kDa). They stated that the nucleic acid transfection efficiency, cytotoxicity, and tumor growth inhibition ability of the combination strongly depend on the micelle's molecular architecture (Qian et al., 2014). Another used dendrimer for its proton sponge effect is PAMAM, as reported in the work of Ren et al., where they designed hollow gold nanoparticles attached with thiolated PAMAM through the strong Au—S linkage. Upon cellular entry, the dendrimer could escape the endosomes. Afterward, the miRNA molecules were released into the cytoplasm, modulating the intrinsic state of cancer cells and cancer stem cells (CSCs) to a more chemosensitive state to Doxorubicin treatment (Ren et al., 2016).

#### 3.5.2 With helper lipids

Neutral helper lipids such as (1,2-dioleyl-sn-glycerol-3-phosphoethanolamine) DOPE, which is included in many lipid-based nanoparticles, can disturb the endo/lysosomal membrane integrity (Sankhagowit et al., 2016). This effect was well described by Feng et al. They designed a liposome with a shell containing a mixture of cationic lipids and DOPE. Their nanoparticles showed a rapid endosomal/lysosomal escape after 4 h of incubation due to endo/lysosomal membrane disturbances mediated through the lipid DOPE. DOPE facilitated liposome fusion with the endo/lysosomal membrane through the formation of a hexagonal inverted phase structure in the acidic environment of the endo/lysosomal compartment. This transformational change enabled endo/lysosomal membrane destabilization processes (Feng et al., 2014).

#### 3.5.3 With endosomal osmotic pressure increment

Increasing the osmotic pressure inside the endosomes can be achieved through the chemical generation of byproducts inside the cells, for example, by drug release. He et al. developed nanoscale coordination polymer-loaded siRNA and CisPt. After the cells had uptaken the nanoparticles, the release of CisPt *via* reductive cleavage of the metal-ligands bonds was associated with generating two molecules of CO_2_. The gas liberation led to osmotic pressure change inside the endosomes, further disturbing the endosomal membrane and promoting siRNA endosomal escape into the cytosol (He et al., 2016). An increase in the osmotic pressure inside the endosomes could also be induced using calcium phosphate (CaP). Zhang et al. developed Lipid/calcium/phosphate (LCP) nanoparticles to co-deliver c-Myc siRNA and GEM. At the low pH of endosomes, CaP is dissolved, leading to osmotic pressure increment inside the endosome. This osmotic pressure increment finally resulted in endosome rupture, releasing the entrapped c-Myc siRNA and GEM into the cytoplasm. Furthermore, this release could be assured by the destabilizing action mediated by the cationic lipid 1,2-Dioleoyl-3-trimethylammonium propane (DOTAP) that coated the LCPs. Cationic DOTAP interacted with the endosome anionic membrane, disturbing the membrane structure even more (Zhang et al., 2013).

### 3.6 Efficient therapeutics release

It is important to consider drug release when developing a nanoparticle delivery system. Efficient drug release from the nanoparticles to the targeted machinery is triggered either by chemical reactions or disturbances in the thermodynamic balance of the loaded drug in response to many stimuli. The drug release rate generally depends on drug solubility, desorption of the surface-bound or adsorbed drug; drug diffusion through the nanoparticle core; nanoparticle core degradation; and the combination of degradation and diffusion processes (Hoffman, 2008).

Covalently loaded drugs might need bond cleavage (*i.e.*, photo-triggered release, thermal bond cleavage, bond reduction…*etc.*), while non-covalently loaded drugs on the surface of NPs require a change in local physical forces. The non-covalently loaded drugs into the nanoparticles are easily released, but they can be uptaken by non-specific cells. In contrast, many covalently bound systems have a low off-target release but require an external stimulus (*i.e.*, high concentrations of enzymes, light, pH, thermal energy…*etc.*) to release the loaded therapeutics. This issue is well discussed in the review of Doane and Burda (2013).

The information about the differences between tumor microenvironment and normal cells inspired the design of many smart nanoparticles. Information about tumor microenvironment and intracellular signal-activated nanomaterials was amazingly illustrated in a review presented by Mo and Gu (2016). The most important thing for the co-delivery of nucleic acids and anti-cancer drugs is ensuring the therapeutics reach the desired concentrations in tumors. This synchronizing can be attained by reducing the therapeutic leakage from the applied nanoparticles, reducing the premature inactivation of the drugs during circulation, or increasing the drug release at the tumor sites. To achieve selective drug release at tumor sites; pH/redox or ATP-sensitive moieties were used, and for further control of the release, stimuli-responsive features are applied, as illustrated in Table 3.

**Table 3:** Mechanisms and moieties involved in stimulus-responsive therapeutics release in nanoparticles mediated combinational therapy.

Name of the moiety	NPs type	Loaded Therapeutics	Mechanism involved in the therapeutic release	References
**Redox-sensitive release**				
Imidazolium	Liposomes	siRNA/Paclitaxel	Imidazole ring protonation	Reddy et al., 2016
Oligopeptide lipid (LHSSG2C14)	Liposomes	siRNA/Paclitaxel	Lysine and histidine protonation	Chen et al., 2017b
Poly (β-amino esters)	Hollow Mesoporous Organosilica Nanoparticles	siRNA/DOX	Poly (β-amino esters) protonation	Wu et al., 2016
Cystamine	PEI based Nanoparticles	pDNA/DOX	Disulfide bond cleavage	Davoodi et al., 2016
Cystamine	Polymeric micelles	siRNA/Paclitaxel	Disulfide bond cleavage	Zhu et al., 2017
Linear decanoyl chain	PLGA based Nanoparticles	siRNA/Cisplatin	Reductive linker cleavage	Xu et al., 2013b

**pH-sensitive release**				
Glutamic acid	Dendrimer	pDNA/DOX	Hydrazone bond cleavage	Liu et al., 2012
Poly aspartyl (N- (N′, N′-diisopropylaminoethyl))	Polymeric micelles	siRNA/DOX	Protonation	Sun et al., 2018

**Dual redox/pH-sensitive release**				
Silsesquioxane	Silica based Nanoparticles	pDNA/DOX	Disulfide bond cleavage	Zhang et al., 2017
Poly (acrylhydrazine)	Micelleplexes	siRNA/DOX	Disulfide linkage and pH-sensitive hydrazone bond cleavage	Suo et al., 2017
Polyhistidine	Polyplexes	siRNA/DOX	Polyhistidine protonation and the disulfide bond cleavage.	Gao et al., 2019

**Light-sensitive release**				
Gold	Hollow gold NPs	miRNA/DOX	NIR triggered nanoparticle degradation for DOX release	Ren et al., 2016

**ATP-sensitive release**				
ATP aptamer duplex	PEI based NPs	miRNA/DOX	ATP triggered release	Wang et al., 2017

**ATP:** Adenosine triphosphate, **DOX:** Doxorubicin, **LHSSG2C:** Ditetradecyl 2-(4-(2-(2-(2-(2-(2,6-diaminohexanamido)-3-(1H-imidazole-4-yl) propanamido) ethyl) disulfanyl) ethylamino)-4-oxobutanamido) pentanedioate, **miRNA:** Micro ribonucleic acid, **NIR:** Near infra-red, **NPs:** Nanoparticles, **pDNA:** Plasmid deoxyribonucleic acid, **PEI:** Polyethylenimine, **siRNA:** small interfering ribonucleic acid.

For example, Ren et al. synthesized HGNPs with attached PAMAM dendrimers loading miR-21 and DOX. Inside the cells, the PAMAM moiety electrostatically loaded miRNA mediated its endosomal escape *via* a proton sponge effect. After 4 h, in response to NIR laser stimuli absorption, HGNPs underwent shape transformation and size reduction, leading to burst release of DOX. The release was achieved within 15 min after NIR exposure. The data described that sequential release of anti- miR-21 followed by a burst release of DOX produced a synergistic effect with superior anti-cancer efficacy compared to simultaneous co-delivery (Ren et al., 2016). In the same context, Gao et al. developed polyplexes that release siRNA and Doxorubicin in a synchronized manner by dual redox/pH stimuli. The polyplex consisted of a pH-sensitive PEG-b-PLA-PHis linked with OEI *via* a redox cleavable disulfide bond. The amphiphilic copolymer self-assembled into micelles with a hydrophobic core for DOX encapsulation. The shell of the micelles contained cationic OEI, which was able to form siRNA polyplexes. The authors showed that polyplexes at an N/P ratio of 7 showed more efficient pH/redox triggered the release and more effective *endo*-lysosomal escape of the payloads than the polyplexes at higher N/P ratios, and thereby higher intracellular payload delivery efficiency. This optimized ratio was attributed to the more effective endo-lysosomal escape facilitated by the redox potential cutoff OEI blocks from the copolymer, which permeabilized the endosomal membrane. The increase in the N/P ratio led to a strong electrostatic interaction between siRNA and OEI which resulted in the triggered release of payloads and subsequent endo-lysosomal escape being impeded (Gao et al., 2019).

### 3.7 Additional theranostic properties

Additional theranostic features could be interesting and add value to the designed nanoparticles. The use of contrast agents or specific elements with imaging properties in dual nanotherapeutics is described in the following examples. These imaging agents help monitor the nanosystems' fate *in vitro* and *in vivo*.

Lipiodol® is an iodinated derivative of poppy seed oil that serves as a radio-opaque contrast agent. It is indicated for selective hepatic intra-arterial use for imaging tumors in adults with known hepatocellular carcinoma and trans-arterial chemoembolization (TACE) (Dioguardi Burgio et al., 2019; Miszczuk et al., 2020). Lipiodol® is also used in lymphangiography (imaging of the lymphatic system) (Pieper et al., 2019). Moreover, it has the ability to solubilize many lipophilic drugs. Oh et al. reported the use of Lipiodol®, which was incorporated during the emulsification step into their nanoemulsion. Lipiodol® was used in this study as a contrast agent to solubilize Paclitaxel. The micro-computed tomography (CT) images obtained from *in vivo* models concluded the usefulness of Lipiodol® as a potential contrast agent. The cells transfected with controls were not visualized in nude mice, whereas a bright spot was detected in the mouse injected with the Lipiodol® containing nanoemulsion (Oh et al., 2013). Iron-based nanoparticles can also be used for their theranostic properties. Yu et al. designed iron nanoparticles that loaded both siRNA and CisPt prodrug. These nanocarriers possess imaging properties when placed in a magnetic resonance (MR) field. This property was not altered after the therapeutics loading on the iron-based vector. The *in vitro* magnetic resonance (MR) images showed a concentration-dependent transverse relaxation time (*T*_*2*_) MRI contrast effect. They concluded that Fe_3_O_4_-based nanocarriers had an apparent contrast enhancement in the tumor sites, which could help to visualize the tumor site location, and drug accumulation and enhance the future anti-cancer effect of the payloads accordingly (Yu et al., 2018). Majumder and Minko developed multifunctional lipid-based nanoparticles that co-deliver both epidermal growth factor receptor (EGFR) siRNA and PTX or Gefitinib. Fluorophore rhodamine, which acts as an imaging agent, was loaded into the lipid phase during the synthesis process of the nanoparticles. The images showed that the rhodamine-labeled nanoparticles containing anticancer drugs and siRNA penetrated the lung cancer cells and localized in the cytoplasm, indicating successful cellular uptake of nanoparticles (Majumder and Minko, 2021).

## 4 Biological evaluation of the co-delivered therapeutics for chemoresistance reversal

Chemoresistance status refers to any cellular changes that render the cancer cell incapable of responding to the cytotoxic effect of different chemotherapies. The growing understanding of the mechanism of action of drugs and the ways to acquire resistance with a recent advance in cancer cell complex biology led to a better evolutional therapeutic outcome. The enhanced therapeutic outcomes using combination therapy loaded nanoparticles are observed with the help of resistant cancer cell lines and include a marked increment in anti-cancer drug cellular concentration and localization and/or cell death concerning the used target. Not only the design of the nanoparticles but also the choice of the type of nucleic acid, its sequence, and the associated chemotherapeutic drug are important. Therefore, the basics of nucleic acid selection, the target genes responsible for the MDR phenomenon, and considerations of combining chemotherapeutic drugs and nucleic acids are highlighted in the following part. Examples using different types of nanocarriers to co-deliver nucleic acids and chemotherapeutic drugs are chosen to illustrate the therapeutic outcomes of different combination therapies.

### 4.1 Nucleic acids types for chemoresistance reversal

According to the type of nucleic acids used, specific genes can be restored, overexpressed, or silenced. Plasmid DNA (pDNA) is used to restore or enhance the encoded protein's function and involves the activation of various cellular pathways. For example, introducing wild-type p53 into cancer cells expressing the mutated p53 gene enhances cell chemosensitivity partly by regulating P-gp expression. Besides, the restoration of the functionality of the p53 tumor-suppressing effect, which in turn renders the tumor cells more vulnerable to the DNA damage caused by anti-cancer drugs and regulate various apoptotic pathways (Hientz et al., 2017).

In contrast to pDNA, short interfering RNA (siRNA), short hairpin RNA (shRNA), and micro-RNA (miRNA) mediate their action through the RNA interference mechanism, as mentioned earlier. siRNA are widely used to inhibit the synthesis of specific proteins. In general, siRNA is directly delivered into the cytoplasm, where they bind to the RNA-induced silencing complex (RISC) that will finally lead to complementary mRNA degradation. Most studies we review here used anticancer drugs in combination with siRNA. One example is the study by Chen et al. that used 5-FU in combination with HIF1-α siRNA in order to downregulate HIF1- α and increase the intracellular 5-FU concentration (Chen et al., 2017a).

shRNAs are generally continually expressed inside the cell after transfection of shRNA-plasmids containing the specific RNA sequence and have a long-term effect on knocking down overexpressed genes (Acharya, 2019). Compared to siRNA, shRNAs are processed by Dicer (an RNAse III enzyme) before binding to RISC (Singh et al., 2011). Compared to siRNA, this leads to a more remarkable ability to bind RISC and slow intracellular metabolic rate (Acharya, 2019; Taxman et al., 2010). An example of combination therapy with shRNA is the work of Babaei et al., in which survivin shRNA expressing plasmid DNA combined with Camptothecin was employed (Babaei et al., 2020).

miRNAs are also processed by RISC and can induce either mRNA degradation or inhibit the mRNA translation due to imperfect complementarity (Singh et al., 2011). Therefore, miRNA can target and regulate the expression of multiple genes – at the transcriptional and translational level – in different cellular pathways (Oliveto et al., 2017). The combined treatment of chemotherapeutics and miRNA has been proven to be a viable strategy for enhancing chemosensitivity due to its synergistic effect on tumor therapy *via* different pathways, either through targets affecting cellular drug accumulation or through alteration of compensating mechanisms including pro/anti-apoptotic pathways, DNA repair mechanism, autophagy, invasion, migration, and metastasis involved in chemoresistance as presented in Table 4. To enlighten the complexity of miRNA delivery and its regulatory effects, we recapitulate hereafter a study performed by Ren et al. (Ren et al., 2016). They utilized an exciting, detailed approach for miR-21 inhibition in breast cancer cells and cancer stem cells (CSC). In this context, they developed a hollow gold nanoparticle (HGNP), combined with anti-miR-21 and DOX to be released sequentially. Firstly, anti-miR-21 was allowed to regulate genes of interest; then, within 4 h, DOX was released in response to NIR laser exposure (Ren et al., 2016). The nanoparticle uptake by the cells was up to 100%, and miR-21 expression was inhibited by 60%. Cell toxicity values of free DOX, simultaneous and sequential co-delivery of DOX and miR-21, were 29%, 45%, and 69%, respectively. DOX treatment on MDA-MB-231 cells gave an IC_50_ value of 1.1 μg/mL, which decreased to 0.27 μg/mL, and 0.13 μg/mL when DOX was used as simultaneous combined delivery and sequential combined delivery, respectively. In this cell line, the sequential combined delivery exhibited an 8-fold reduction rate, while on MCF-7, the same treatment reduced the IC_50_ by 12-folds. The apoptotic cells gradually increased in MDA-MB-231 cells from 7.9 ± 0.8% to 21.6 ± 1.3% and 32.5 ± 2.4%, and in MCF-7 from 6.0 ± 0.7% to 15.8 ± 1.4% and 25.5 ± 1.8%, using free DOX, co-delivery, and sequential co-delivery, respectively. This interesting synergistic activity was evaluated by the combination efficacy (Q). If Q > 1.15, it means that there is a synergism between the two treatments. If 0.85 < Q < 1.15, there is an additive effect, while if Q < 0.85, it indicates an antagonism. Within 5-days using either the co-delivery or sequential co-delivery, the Q values were 0.76–1.24 indicating a moderate synergistic effect for co-delivery, while for sequential co-delivery, the Q value was 1.87, indicating an enhanced synergistic effect. This enhanced cell death was achieved by activating different cell death pathways *via* inhibiting miR-21 expression. Definitely, miR-21 inhibition led to an increased level of cytochrome *c* and apoptosis-inducing factor (AIF), along with the increased expression of p53, Bax, cleaved caspase-3, and caspase-9. Moreover, Bcl-2 protein expression reduced the activated mitochondrial apoptosis pathway. Also, Fas ligand (FasL) and cleaved caspase-8 expression increased, leading to the activation of the death-receptor-mediated apoptosis pathway. To reverse stemness in CSC, miR-21 downregulated the three stem cell-specific transcriptional factors Nanog, Oct4, and Sox2. CSC collected from MDA-MB-231 cells were isolated and treated with HGNP. The results showed that sequential treatment release modulated the stemness of CSC to a more chemosensitive state, which enhanced the cellular uptake of DOX. The IC_50_ of free DOX in CSC was 20.7 μg/mL, dramatically decreasing to 1.3 μg/mL using the co-delivery and to 0.39 μg/mL using the sequential co-delivery. Lastly, *in vivo* studies revealed that the sequential co-delivery showed the highest tumor growth inhibition compared to free DOX or the co-delivery (Ren et al., 2016).

**Table 4:** Reversal of chemoresistance through micro RNAs.

miRNA	Anticancer drug	Downstream genes regulation	Biological impact	References
**Targets affecting drug accumulation**				
miR-129-5p	Doxorubicin	↓ P-gp ↓ CDK-6	↑↑ Drug accumulation ↑↑ Cell cycle arrest	Yi et al., 2016

**Alteration of compensating mechanism involved in chemoresistance**				
anti-miR-21	Doxorubicin	↓ Bcl-2 ↓ PTEN and pAKT ↑ Caspase-3	↑↑ Apoptosis ↓↓ Cell survival pathway	Qian et al., 2014
miR-34a	Doxorubicin	↓ Bcl-2 ↓ Notch-1 pathway	↑↑ Apoptosis ↓↓Migration and metastasis	Deng et al., 2014
miR-205	Gemcitabine	↓ ZEB, SIP-1, HRAS, and LRP ↑ *E*-CAD and CAV-1	↑↑Apoptosis ↓↓ Invasion and migration	Mittal et al., 2014
miR-212	Doxorubicin	↓ USP9X and vimentin	↑↑Apoptosis and autophagy	Chen et al., 2019
anti-miR-221/222	Paclitaxel	↑ p27^Kip1^ and TIMP3	↑↑Apoptosis ↓↓ Metastasis	Zhou et al., 2017
miR-375	Cisplatin	↑ Bax and Caspase-3 ↓ Bcl-2	↑↑ Apoptosis	Yang et al., 2016

↓**:** Gene expression downregulation, **↑:** Gene expression upregulation, **↑↑:** increase or induction, **↓↓:** inhibition or reduction.

**P-gp:** P-glycoprotein, **Bcl-2:** B-cell lymphoma-2 antiapoptotic protein, **PTEN:** Phosphatase and tensin homolog tumor suppressor gene, **pAKT:** Phosphatidylinositol 3-kinase and Protein Kinase survival pathway, **Notch-1:** Notch homolog 1, translocation-associated, **ZEB:** Zinc finger E-box-binding homeobox 1 transcription factor, **SIP-1:** Stress-induced protein **HRAS:** GTPase HRas enzyme, **LRP:** Lung resistant protein, **E-CAD:** E-cadherin, **CAV-1:** Caveolin 1, **USP9X:** ubiquitin-specific peptidase 9, X-linked, **CDK-6:** Cyclin-dependent kinase-6, **p27**^**Kip1**^**:** Cell cycle inhibitor, **TIMP3:** Tissue inhibitor of metalloproteinase 3, **Bax:** Bcl-2-associated X protein apoptotic activator.

### 4.2 Chemoresistance reversal mechanisms and corresponding molecular targets

In this part, the focus is on the major mechanisms to reverse chemoresistance, which include the regulation of (i) genes that are responsible for anti-cancer drug internalization and its cellular concentration, (ii) genes involved in DNA repair mechanism, and (iii) genes implicated in apoptotic pathways. Examples are given using the different types of nucleic acids mentioned before.

#### 4.2.1 Improvement of intracellular drug accumulation

Decreased intracellular levels of cytotoxic agents are one of the most common manifestations of drug resistance. Enhancing intracellular drug accumulation can be maintained by controlling angiogenesis. Examples of combination therapy can be found in the literature for this approach using, for example, anti-VEGF (Vascular endothelial growth factor) siRNA (Feng et al., 2014) or downregulating the hypoxia-inducible factor 1 (HIF1), which is one of the master transcriptional regulators of cellular angiogenesis (Zhao et al., 2015; Chen et al., 2017a).

Chen et al. reported that downregulation of HIF1α by siRNA increased 5-FU accumulation in human gastric cancer cell line SGC-7901 by inhibiting P-gp efflux. Moreover, it could sensitize 5-FU/resistant cells to the action of 5-FU and decrease the resistance index (R). To achieve that, they utilized chitosan-based nanoparticles to efficiently deliver therapeutics *in vitro* and *in vivo*. The IC_50_ of 5-FU was 371.73 μg/mL in SGC-7901/5-FU resistant cells, with an R-value of 9.8. The combination induced HIF1-α downregulation at the protein and mRNA levels. Consequently, the results suggested that HIF1α siRNA can reduce P-gp efflux by down-regulating HIF1α expression. Moreover, it decreased the IC_50_ significantly (*P* < 0.01) compared to the group treated with 5-FU alone, and R-value decreased to 6.6 in 5-FU resistant cells. Mice treated with the combination had a significant tumor growth inhibition compared to mice treated with monotherapies, without any apparent toxicity (Chen et al., 2017a).

Efflux pump transporters increase the drug cell detoxification process by increasing drug efflux, decreasing drug uptake, and inducing drug structural modifications. Inhibiting this transporter system using siRNA or miRNA will also restore the therapeutic anti-cancer drug concentration. Examples in the literature of combination therapy targets, for example, ATP-binding cassette Subfamily B Member 1 (ABCB1), also known as multi-drug resistant gene 1 (MDR-1) or P-glycoprotein P (P-gp) (Zhang et al., 2016), and ABCG2 also known as breast cancer resistant protein BCRP (Zhu et al., 2017).

Zhu et al. described the targeting of ABCG2 protein using siRNA by their polymeric micelles. This approach aimed to inhibit MDR by silencing the ABCG2 gene, restoring PTX intracellular concentration, and maximizing its therapeutic effect on MCF-7 and MCF-7/Taxol resistant human breast cancer cell lines. They concluded that siRNA reduced the expression of efflux proteins and maintained a high intracellular concentration of PTX to induce cytotoxicity. Cells treated with micelles containing ABCG2 siRNA had >90% inhibition of ABCG2 expression at the mRNA level, and approximately 80% of ABCG2 protein expression was inhibited. Interestingly, the combination (anti-ABCG2 siRNA + PTX) had the best synergistic anti-tumor effect among all the tested formulations proving reversal of MDR (Zhu et al., 2017).

Another interesting mechanism involves controlling the vault proteins, which are responsible for the cytotoxic drug uptake in the cytosol and sequestration of the drugs into exocytosis vesicles (Dalton and Scheper, 1999; Zurita et al., 2003). Consequently, knockdown of the vault proteins encoded genes using siRNA will facilitate drug exposure to its cellular targets. One example is using combination therapy to target the major vault protein (MVP), also known as lung resistant related protein (LRP) (Han et al., 2012).

#### 4.2.2 Inhibition of DNA repair mechanisms

Most chemotherapeutic drugs cause DNA damage to exert their cytotoxic effect. DNA damage leads to the activation of several cellular pathways, such as DNA repair pathways, to remove the damaged DNA and promote translesion DNA synthesis (TLS). TLS is a DNA damage tolerance process that allows the DNA replication machinery to replicate past DNA lesions. That, in turn, enhances cancer cells' capacity to repair or tolerate DNA damage, resulting in acquired chemoresistance. The DNA repair protein reversionless 1 (REV1) is a translesion DNA polymerase, while the protein reversionless 3-like (REV3L) is a catalytic subunit of the translesion DNA polymerase (Polζ), and REV7 is the auxiliary subunit. REV3L/REV7 was shown to play a role in chemoresistance to many drugs acting on DNA like Cyclophosphamide and Cisplatin (Xu et al., 2013b).

Anti-REV1 and REV3L siRNA combined with Cisplatin prodrug co-delivery in PLGA-based nanocarriers was evaluated by Xu et al. in the human prostate cancer cell line LNCaP *in vitro* and *in vivo* and on the breast cancer cell line MDA-MB-231 *in vitro*. The combined delivery inhibited TLS activity, impaired drug-induced mutagenesis, and consequently sensitized cancer cells to the DNA-damaging effect of Cisplatin. The co-delivery achieved sustained and reduced REV1/REV3L protein expression up to 87% *in vitro*, and up to 78% *in vivo*, over 3 days. Besides, the combination had a more significant induction of Cisplatin chemo-sensitization, indicated by a lower value of IC_50_, compared to individual agent treatments. Mice treated with intratumoral injection had the highest tumor inhibition, with 50 days of survival without tumor growth, compared to mice treated with PBS or monotherapies (Xu et al., 2013b).

Another approach involved regulating the Ras-related C3 botulinum toxin substrate 1 (Rac1) gene. This gene activates the non-oxidative pentose phosphate pathway and simultaneously enhances the nucleoside metabolism *via* activating aldolase and extracellular-signal-regulated kinase (ERK) pathways. Upregulating the ribose 5 phosphate (R5P) synthesis and nucleoside metabolism, thus promoting the repair of DNA damage caused by chemotherapeutic agents and inducing chemoresistance to these drugs (Kazanietz and Caloca, 2017).

Li et al. utilized anti-Rac1 siRNA and Cisplatin within endosomal pH-responsive nanoparticles. The combination was tested in a patient-derived xenografts (PDX) mouse model from neoadjuvant chemotherapy-resistant breast cancers. Systemic knockdown of the Rac1 gene increased the sensitivity of breast tumors to chemotherapies. Co-delivery of anti-Rac1 siRNA and Cisplatin led to the total regression of tumors in some mice, indicating a dramatic synergistic effect compared to either anti-Rac1 siRNA or Cisplatin delivery alone. Ki67 and cleaved Caspase-3 staining results confirmed both decreased proliferation and increased apoptosis in the xenograft, respectively. The IC_50_ doses of siRac1 NP, Cisplatin NP, and the concentration of anti-Rac1 or Cisplatin in siRNA/Cisplatin NP that provided the same effect were examined in MDA-MB-231 cells, respectively. The combination index was found to be 0.69 (a value of less than one indicating a synergistically inhibitory effect on tumor cell growth). These findings conclude that the pH-responsive nanoparticle co-encapsulated and co-delivered anti-Rac1 siRNA and cisplatin provided a promising strategy to sensitize breast cancer to the DNA damaging effect of chemotherapeutic agents (Li et al., 2020).

#### 4.2.3 Cell death induction by modulating apoptotic pathways

Cytotoxic agents act by a wide variety of pathways to induce selective tumor cell death. The response of chemotherapeutic drugs mainly depends on the direct cell damage and on the tumor cells' capacity to respond to these harms by inducing the apoptotic machinery. Therefore, the drug's effect is associated with the expression of specific genes of anti/pro-apoptotic pathways and survival pathways (Fodale et al., 2011). Down/up-regulation of former pathways will result in better therapeutic outcomes and reversal of chemoresistance through the regulation of compensating mechanisms responsible for cell death.

siRNA and shRNA can be used to downregulate specific anti-apoptotic proteins. Examples of combination therapies target for example survivin (Babaei et al., 2020), Bcl-2 (B-cell lymphoma 2), (Oh et al., 2013), c-Myc (c-master regulator of cell cycle entry proliferation, and metabolism, Zhang et al., 2013), Metadherin (MTDH, Yang et al., 2018), STAT-3 (Signal transducer and activator of transcription-3), (Su et al., 2012), AKT-1 (Serine/Threonine Kinase-1), (Guo et al., 2012), FAK (Focal adhesion kinase) (Byeon et al., 2018), and Kras (Kirsten rat sarcoma viral oncogene homolog) (Wen et al., 2017).

Yang et al. combined anti-MTDH siRNA and PTX using PLGA-based nanoparticles and studied the relation between MTDH expression and PTX effectiveness in the human breast cancer cell line MCF-7. They showed that MTDH overexpression increased tumor growth and decreased PTX treatment efficacy. Also, they described that MTDH overexpression increased protein expression of p65 and phosphor p65 (p-p65) – subunits of the nuclear factor kappa-light-chain-enhancer of activated B cells (NF-κB) transcription factor family and reduced the expression of IκBα (inhibitor of NF-κB, alpha). NF-κB is a complex protein that controls DNA transcription, cytokine production, and cell survival. NF-κB pathway regulates anti-apoptotic genes and caspase action. Thus, targeting MTDH would negatively impact the NF-κB pathway, increasing PTX sensitivity. The loaded nanoparticles downregulated MTDH expression both *in vitro* and *in vivo*. Due to MTDH downregulation, the combination induced the highest rate of cell death compared to all other formulations that used monotherapies. Moreover, mice treated with the combination experienced neither weight loss nor tissue damage caused by free PTX treatment (Yang et al., 2018).

Furthermore, plasmid DNA can be used to upregulate pro-apoptotic proteins such as p53 (Liang et al., 2015) or tumor necrosis factor-related apoptosis-inducing ligand (TRAIL) (Liu et al., 2012). Liang et al. combined wild-type p53 plasmid DNA with DOX in calcium carbonate/calcium phosphate-based nanoparticles to perform *in vitro* cytotoxicity studies on HeLa (human cervical cancer) cell line. HeLa cells treated with the combination had the highest dose-dependent cell inhibitory effect, concordance with DOX increment among the used formulations as free DOX and DOX encapsulated NPs. Besides, in the cell morphology studies, 48 h post-combination treatment, the cells appeared round with visible blebbing and reduced number as an indication of apoptosis induction. All these findings proved the efficiency of p53 introduction to enhance the chemosensitivity of cells to the anti-cancer effect of DOX (Liang et al., 2015).

### 4.3 Efficient combinations of nucleic acids with chemotherapeutics for chemoresistance reversal

This section presents considerations regarding the mechanisms of action of the chemotherapeutic drugs and the molecular targets of nucleic acids for efficient chemoresistance reversal and chemotherapeutic action. Some examples are detailed in this section. More examples with the corresponding references can be found in Table 5.

**Table 5:** Summary of anti-cancer drugs combined with nucleic acids delivered by various types of nanoparticles for chemoresistance reversal.

Biological action	Nucleic acids type	Target	NPs type	Type of cancer	Ref.
**Doxorubicin**					
Enhancement of drug accumulation	siRNA/shRNA	P-gp	Polymeric micelles	Breast cancer	Shen et al., 2014
				Hepatocellular cancer	Zhang et al., 2016
			Micelle-like NPs	Breast cancer	Navarro et al., 2012
			PAMAM-based NPs	Breast and ovarian cancer	Pan et al., 2019
			Silica-based NPs	Breast cancer	Meng et al., 2013
					Wu et al., 2016
			pH/Redox responsive polyplexes	Breast cancer	Gao et al., 2019
		ABCB1/ABCG2/ ABCC1	Carbonate apatite NPs	Breast cancer	Tiash and Chowdhury, 2019
		LRP	PAMAM-based NPs	Breast cancer	Han et al., 2012
		VEGF	Liposomes	Ovarian cancer	Chen et al., 2010b
	miRNA	miR-129-5p	Peptide-based NPs	Breast cancer	Yi et al., 2016
Regulation of apoptotic pathways	siRNA/shRNA	Bcl-2	Polymeric micelles	Breast cancer	Suo et al., 2017
				Hepatocellular cancer	Sun et al., 2018
			PEI-based NPs	Lung cancer	Xu et al., 2015
			Silica-based NPs	Breast cancer	Zhou et al., 2016
				Ovarian cancer	Chen et al., 2009
			Quantum dots	Breast cancer	Yue et al., 2020
		Bcl-xl	PLGA-based NPs	Breast cancer	Ebrahimian et al., 2017
			Polymersomes	Gastric cancer	Kim et al., 2013
		IL17 B	Chitosan-based NPs	Breast cancer	Alinejad et al., 2016
		Survivin	Silica-based NPs	Hepatocellular cancer	Li et al., 2017
		Kras	Polyjuglanin NPs	Lung cancer	Wen et al., 2017
		c-Myc	Liposomes	Ovarian cancer	Chen et al., 2010a
		Beclin 1	OEI-based NPs	Ovarian cancer	Jia et al., 2015
	miRNA	miR-34a	Chitosan-based NPs	Breast cancer	Deng et al., 2014
			PEI-based NPs	Lung cancer	Wang et al., 2017
		miR-212	Peptide-based NPs	Pancreatic cancer	Chen et al., 2019
		miR-21	Hollow gold NPs	Breast cancer	Ren et al., 2016
	pDNA	*p53*	PLGA-based NPs	Hepatocellular cancer	Xu et al., 2013a
			Cyclodextrin-based NPs	Breast cancer	Lu et al., 2011
			Silica-based NPs	Glioma	Zhang et al., 2017
			Calcium phosphate NPs	Cervical cancer	Liang et al., 2015
			Alginate/CaCo_3_ hybrid NPs		Zhao et al., 2012
			Solid lipid NPs	Lung cancer	Han et al., 2014
		TRAIL	PEI-based NPs	Hepatocellular cancer	Davoodi et al., 2016

**Paclitaxel**					
Enhancement of drug accumulation	siRNA/shRNA	ABCG2	Polymeric micelles	Breast cancer	Zhu et al., 2017
Regulation of apoptotic pathways		Bcl-2	Liposomes	Melanoma	Reddy et al., 2016
			Nanoemulsion	Breast cancer	Oh et al., 2013
		Survivin	Polymeric micelles	Breast cancer and ovarian cancer	Salzano et al., 2015
			Liposomes	Breast cancer	Chen et al., 2017b
			Polymeric micelles	Ovarian cancer	Hu et al., 2012
			Peptide-based NPs	Lung cancer	Shen et al., 2014
		TLR4	Polymeric micelles	Ovarian cancer	Jones et al., 2016
		MTDH	PLGA-based NPs	Breast cancer	Yang et al., 2018
		FAK		Ovarian cancer	Byeon et al., 2018
		STAT-3		Lung cancer	Su et al., 2012
		AKT-1	Poloxamer hydrogel	Breast cancer	Guo et al., 2012
		EGFR	Multifunctional lipid-based NPs	Lung cancer	Majumder and Minko, 2021
	miRNA	miR-221/222	Calcium phosphate-polymer hybrid nanoparticles	Breast cancer	Zhou et al., 2017
	pDNA	TRAIL	Liposomes	Glioma	Sun et al., 2011
		Wild type p53	Cyclodextrin-based cationic polymers	Lung cancer	Zhao et al., 2014

**Docetaxel**					
Regulation of apoptotic pathways	siRNA/shRNA	Bcl-2	Peptide-based NPs	Breast cancer	Zheng et al., 2013
			Liposomes	Lung cancer	Qu et al., 2014

**Cisplatin**					
Regulation of apoptotic pathways	siRNA/shRNA	Bcl-2 siRNA	Polymeric micelles	Breast cancer	Xiao et al., 2017
		Survivin/Bcl-2	Hyaluronic acid-based NPs	Lung cancer	Ganesh et al., 2013
			Nanoscale coordination polymer	Ovarian cancer	He et al., 2016
		EZH2	Iron-based NPs	Ovarian cancer	Yu et al., 2018
	miRNA	miR-375	Lipid-based NPs of Cisplatin	Hepatocellular cancer	Yang et al., 2016
Regulation of DNA repair mechanism	siRNA/shRNA	REV1/REV3L	PLGA-based NPs	Prostate cancer	Xu et al., 2013b
		Rac1	Endosomal pH-responsive NPs	Breast cancer	Li et al., 2020

**Gemcitabine**					
Enhancement of drug accumulation	siRNA/shRNA	HIF-1α	Lipid polymer hybrid NPs	Pancreatic cancer	Zhao et al., 2015
Regulation of apoptotic pathways	miRNA	c-Myc	Calcium phosphate-based NPs	Lung cancer	Zhang et al., 2013
		miR-205	Polyplexes	Pancreatic cancer	Mittal et al., 2014

**5-Fluorouracil**					
Enhancement of drug accumulation	siRNA/shRNA	HIF-1α	Chitosan-based NPs	Gastric cancer	Chen et al., 2017a

**Camptothecin**					
Regulation of apoptotic pathways	siRNA/shRNA	Survivin	Mesoporous silica NPs	Colon adenocarcinoma	Babaei et al., 2020

**ABC:** ATP Binding Cassette Subfamily, **AKT:** Serine/threonine Kinase, **Bcl-2:** B-cell lymphoma-2 anti-apoptotic protein, **Bcl-xl:** B-cell lymphoma-extra-large, **c-Myc:** C-Master regulator of cell cycle entry, proliferative and metabolism, **EFGR:** Epidermal Growth Factor Receptor, **EZH2:** Enhancer Of Zeste 2 Polycomb Repressive Complex 2 Subunit, **FAK:** Focal Adhesion Kinase, **HIF1:** Hypoxia Inducible Factor 1, **IL-17B:** Interleukin 17B, **Kras:** Kirsten rat sarcoma GTPase enzyme, **LRP:** Lung Resistant Protein, **miRNA:** Micro ribonucleic acid, **MTDH:** Metadherin, **NPs:** Nanoparticles, **OEI:** Oligoethylenimine, **PAMAM:** Polyamidoamine, **pDNA:** Plasmid deoxy ribonucleic acid, **PEI:** Polyethylenimine, **P-gp:** P-glycoprotein, **PLGA:** Poly (Lactic-*co*-Glycolic Acid), **Rac1:** Ras-related C3 botulinum toxin substrate 1, **REV:** Reversionless phenotype, **shRNA:** Short hairpin ribonucleic acid, **siRNA:** Small interfering ribonucleic acid, **STAT-3:** Signal Transducer and Activator of Transcription 3, **TLR-4:** Toll-Like Receptor 4, **TRAIL:** Tumor necrosis factor (TNF)-Related Apoptosis-Inducing Ligand, **VEGF:** Vascular endothelial growth factor.

#### 4.3.1 Anthracyclines

DOX represent the anthracycline family. DOX resistance is due to multiple reasons. One of them is its low availability to the nucleus. Once in the nucleus, DOX intercalates with DNA and subsequently induces activation of different apoptotic pathways. Interesting combinations with DOX target genes that can increase the cellular concentration of DOX, like P-gp and ABCG2 transporters along with LRB, enhances DOX delivery into the nucleus. Moreover, combination therapies acting on pro-apoptotic p53, a tumor suppressor gene that activates all apoptotic pathways, can potentiate the DOX therapeutic action and reverse its resistance status in cancer cells. Also, DOX could enhance the TRAIL apoptosis induction (Cox and Weinman, 2016; Lin et al., 2018).

Zhang and his group tried to target P-gp using siRNA *co*-delivered with DOX by their polymeric micelles. They reported effective downregulation of P-gp expression and increased intracellular DOX concentration in HepG2/ADM hepatocellular carcinoma cells, a DOX-resistant cell type. *In vitro*, P-gp downregulation improved the therapeutic efficacy of DOX 48 h post-treatment, with a gradual increase of DOX concentration in the nucleus. The IC_50_ for DOX and siRNA-DOX/micelles were 2.24 μg/mL and 9.54 μg/mL after 24 h of treatment in HepG2/ADM cells, respectively. 48 h post-treatment, the IC_50_ decreased remarkably to 1.28 μg/mL and 5.23 μg/mL, respectively, in the same type of cells compared to 24 h treatment. These findings were further confirmed by *in vivo* studies. Indeed, DOX/P-gp siRNA/micelles exhibited the highest anti-tumor activity compared to monotherapies. Moreover, the tumor volume of the mice treated with DOX, siRNA, and siRNA-DOX/micelles were about 500mm^3^, 900mm^3^, and 300mm^3^, respectively, after 24 days of treatment. Post-analysis of P-gp expression from the animal tumors showed a consistent downregulation of P-gp expression (Zhang et al., 2016).

Wen et al. used polyjuglanin nanoparticles to deliver anti-Kras siRNA and DOX to A549/DOX resistant and H69/Cisplatin resistant lung cancer cell lines, *in vitro* and *in vivo*. These combined nanoparticles decreased the Kras protein level in a dose-dependent manner. More interestingly, they decreased MDR-1 and c-Myc protein expression; both contribute to chemotherapeutic drug resistance. Moreover, they increased the p53 expression levels, which act as a tumor suppressor gene. These cellular regulatory effects collectively led to a remarkable *in vitro* cytotoxicity. Besides, they sensitized the cells toward the action of DOX, confirmed by the cell death induction through MTT and colony formation assays. The MTT results in A459/DOX resistant cells showed the restoration of cell DOX sensitivity. Indeed, the DOX-treated cells had >95% viability after 24 h of treatment at a DOX concentration of 10 μg/mL, and viability dropped to ≈40% using the Kras/DOX NPs in the same condition of treatment. The cell death indicated the successful restoration of DOX sensitivity in DOX resistant A549 cell line. Moreover, they also induced efficient suppression of tumor growth *in vivo* (Wen et al., 2017)*.*

#### 4.3.2 Taxanes

Taxanes mainly interfere with microtubule stabilization and different cell death pathways depending on the dose concentration. For example, PTX at high concentration causes mitotic arrest at the G2/M phase, whereas, at low concentration, it induces apoptosis at G0 and G1/S phase either through rapidly accelerated fibrosarcoma-1 (Raf-1) kinase activation or by activation of p53/p21 pathway (He et al., 2005; Yusuf et al., 2003). Also, PTX is considered a substrate for P-glycoprotein. Overexpression of this transport system is recognized as a relevant mechanism of PTX resistance. Additional mechanisms include microtubule changes, cell cycle progression, and regulation of cell death pathways. PTX or DTX are often combined with nucleic acids acting on apoptotic pathways to attain a synergistic activity of the cytotoxic drugs and MDR reversal (Yusuf et al., 2003; Zunino et al., 1999).

Byeon et al. described using Focal adhesion kinase (FAK) siRNA with PTX in hyaluronic acid decorated PLGA nanoparticles to treat drug-resistant ovarian cancer. The combination was tested in chemoresistant epithelial ovarian cancer (EOC) models and a human ovarian patient-derived xenograft (PDX) orthotopic mouse model. The combination induced a significant increase in apoptosis, which was attributed to the fact that FAK silencing led to decreased pAKT expression, which in the end sensitized the cells to the action of PTX. Moreover, the orthotopic mouse model treated with the combination had significant inhibition of tumor growth and a 60% chance of survival for at least 50 days post-treatment, compared to the control and other treatment groups where all the mice died within 45 days. Similarly, in the drug-resistant PDX model, tumor growth was significantly inhibited using combination therapy compared to free PTX or non-targeted PLGA nanoparticles (Byeon et al., 2018).

Because survivin overexpression is related to taxane resistance, as reported by Zaffaroni et al., the development of novel combinations containing both anti-survivin siRNA and taxane has been investigated to evaluate the effectiveness of chemoresistance reversal (Zaffaroni et al., 2002). This combination was well described by Salzano et al. They developed multifunctional polymeric micelles, which combined anti-survivin siRNA and PTX, and they tested it in human ovarian adenocarcinoma resistant cell line, SK-OV-3-tr. This combination resulted in 90% inhibition of survivin protein expression. PTX is well known to reduce survivin expression due to mitosis arrest. *In vivo, the* anti-tumor activity of the combination showed the highest anti-tumor activity with the most negligible toxicity and a complete absence of liver toxicity compared to free PTX. Moreover, the SK-OV-3-tr cells pretreated with anti-survivin siRNA for 48 h followed by a treatment of 40 nM PTX attained cell viability of ≈ 65% 24 h post-treatment compared to the same cells without survivin siRNA pretreatment, which had >90% cell viability. Additionally, the combination restored sensitization of resistant ovarian tumors to low doses of PTX (Salzano et al., 2015).

Zhou et al. developed calcium phosphate-polymer hybrid nanoparticles to simultaneously deliver miR-221/222 inhibitors and Paclitaxel. The combination was tested on the MDA-MB-231 triple-negative breast cancer cell line. Once the MDA-MB-231 cells were treated with the combination, the levels of miR-221/222 were reduced, and hence its subsequent function was inhibited. The inhibition was indicated by the upregulation of p27^Kip1^ and TIMP3, two well-known tumor suppressors. Once the cells restored the tumor-suppressing function of p27^Kip1^ and TIMP3, they retained the therapeutic effect of PTX. The result confirmed the enhanced efficacy of PTX in the combination group tested *in vitro* compared with the groups treated using a free PTX or PTX combined with scrambled miRNA. The combination required only 1% of PTX used in a free form to attain the same 80% cytotoxicity (Zhou et al., 2017).

#### 4.3.3 Platinum-based alkylating agents

Platinum-based drugs are mainly represented by Cisplatin. The mechanism of action of Cisplatin has been associated with its ability to crosslink with the DNA bases to form DNA adducts. These DNA adducts inhibit the repair of the DNA leading to DNA damage and subsequently inducing apoptosis within the cancer cells (Aldossary, 2019). Cisplatin chemoresistance is related to the activation of DNA repair mechanisms, leading to an improved cancer cell tolerance of DNA damage caused by cisplatin. So, the selection of genes involved in the DNA repair mechanism will be ideal for getting the maximum benefits of the combination and restoring the anti-tumor effect of cisplatin.

Furthermore, Yang et al. developed lipid-coated nanoparticles of Cisplatin (NPC) and miR-375. The co-loaded nanoparticles were tested against chemotherapy insensitive hepatocellular carcinoma cell lines HepG2 and Hep3B both *in vitro* and *in vivo*. Up-regulation of miR-375 exerts tumor-suppressing action by targeting Hippo-signaling effector Yes-associated protein (YAP) and astrocyte elevated gene-1 (AEG-1), respectively. Cisplatin induces DNA damage, while miR-375 suppresses the activation of YAP, which helps the cells tolerate the DNA damage. The combined nanoparticles upregulate the expression of Bax and Caspase 3 and downregulate the expression of apoptosis inhibitors Bcl-2 by 60% compared to control groups containing free Cisplatin, NPC, and NPC/miR-375. Both cytotoxicity assays and cell cycle arrest studies confirmed that NPC/miR-375 treatment led to apoptosis of most HepG2 and Hep3B cells. The IC_50_ of cisplatin, NPC, and NPC/miR-375 were 10.4, 3.1, and 0.98 μM, respectively, for HepG2 and 8.8, 3.9, and 1.1 μM, respectively, for Hep3B cells. Moreover, NPC/miR-375-treated HepG2 cells showed a significant percentage of brighter nuclei, indicating a high level of apoptosis in these cells. The cell cycle arrest studies showed that the combination-treated HepG2 were arrested in the G1 phase and could not progress to the S phase. These results verified that NPC/miR-375 had an enhanced cytotoxic effect *in vitro*. On the other hand, the *in vivo* studies revealed that the NPC/miR-375 suppresses tumor growth in a primary hepatocellular carcinoma (HCC) mouse model and a HepG2 xenograft tumor model (Yang et al., 2016).

#### 4.3.4 Pyrimidine antagonist and monoterpene alkaloids

Drug resistance of Gemcitabine involves over expression of drug efflux pumps, nucleotide metabolism enzymes, inactivation of the apoptosis pathway, activation of cancer stem cells, or epithelial-to-mesenchymal transition (EMT) pathway, up/down-regulation of the expression of microRNA (miRNA) (Jia and Xie, 2015). This regulation was illustrated by combinations between GEM and anti-apoptotic c-Myc siRNA or mir-205, which control apoptosis and cancer cell invasion and migration pathways, respectively (Zhang et al., 2013; Mittal et al., 2014). On the other hand, 5-FU showed promising outcomes when it is combined with nucleic acids that target angiogenic growth factors (VEGF) and angiogenesis-relevant genes such as HIF1α and genes associated with the tumor microenvironment (TME) (Chen et al., 2017a; Sethy and Kundu, 2021).

Camptothecin chemoresistance acquisition remains poorly understood but is mainly dependent on its poor cellular accumulation, alteration in the structure of the enzyme topoisomerase I, and alterations in the cellular effect on Camptothecin-DNA complex formation—other shreds of evidence highlight miRNA deregulation. Up to now, combinations are exclusively based on Camptothecin and nucleic acids targeting apoptotic pathways (Beretta et al., 2013). Babaei et al. designed mesoporous silica nanorods (MSNRs) that combine Camptothecin and survivin shRNA expressing plasmid DNA (iSur_pDNA), tested on C26 colon carcinoma cell lines. MTT *in vitro* cytotoxicity results showed that the cell toxicity of the PEG_MSNRs-CPT/Sur significantly increased compared to PEG_MSNRs-CPT, indicating that the down-regulation of survivin expression has sensitized the C26 cells to CPT treatment as an anti-cancer agent. Moreover, *in vitro* apoptotic induction assay results displayed that when cells were treated with PEG_MSNR/Sur or PEG_MSNR-CPT, the percentage of late apoptosis was attained (21.1% and 32%) for PEG_MSNR/Sur and PEG_MSNR-CPT, respectively. In cells treated with PEG_MSNR-CPT/Sur, 58.9% of the late apoptotic population was attained, further confirming the previous finding of a synergistic effect of survivin down-regulation and Camptothecin. *In vivo* anti-tumor efficiency studies confirmed that; the encapsulation of Camptothecin into mesoporous silica nanorods would protect the drug from deactivation, thereby elongating the circulation time of the drug, increasing the cellular uptake, and subsequently increasing its effectiveness as a therapeutic anti-cancer agent. Simultaneous delivery of Camptothecin and survivin shRNA-expressing plasmid causes synergistic therapeutic effects confirmed by the significant reduction of the tumor volume in mice treated with PEG_MSNR-CPT/Sur compared to those treated with either PEG_MSNR-CPT or PEG_MSNR/Sur. The antitumor efficacy of the targeted PEG_MSNR-CPT/Sur was superior among all other treatments (Babaei et al., 2020).

## 5 Conclusion and perspectives

In recent years, research regarding the combination of chemotherapy and nucleic acids has increased at a progressive rate. The primary finding of these studies showed encouraging results for regulating genes responsible for chemoresistance that ultimately lead to the restoration of anti-cancer drug efficacy. This review describes the nanoparticle's primary design considerations and modifications for effective simultaneous delivery with the aim of chemosensitivity restoration. We refer to many technical challenges, beginning from the i) selection of a suitable nanocarrier that can load both cargos with high loading capacities and without the loss of the fundamental nanoparticles integrity and characteristics, ii) avoidance of premature deactivation of nucleic acids by the concurrent loaded anti-cancer drugs, iii) reduction of serum interactions and avoidance of the elimination processes by the mononuclear phagocyte system and the clearance organs, iv) prevention of premature therapeutics release from nanocarrier, and v) specific cellular uptake along with *endo*-lysosomal escape. We recapitulate the main nanocarrier modifications in order to overcome these challenges: separation between the drugs in the cargo, addition of a biocompatible coating, targeting ligands, polymers, and lipids conjugates, and use of stimuli-responsive moieties.

Nevertheless, to optimize the overall anti-cancer effect and minimize the drawbacks of combination-based treatments with the aim of chemoresistance reversal, the biological evaluation of such nanoparticles, designed for a co-delivery of nucleic acids and anti-cancer drugs, is crucial. There is a need for an in-depth understanding of the chemoresistance-related genes and their roles in the evolution of such an unfortunate event to select the right nucleic acid type and sequence. For example, many cytotoxic drugs are considered P-gp substrates, such as taxanes (paclitaxel and docetaxel) the DNA-chelating anthracyclines (doxorubicin and daunorubicin), the topoisomerase inhibitors (camptothecin, topotecan, and etoposide), and others. In this case, nucleic acids inhibiting P-gp can be used to enhance intracellular drug accumulation.

From a clinical point of view, development of combinational modalities between nucleic acid and conventional chemotherapies is in its early stage. All presented treatments were tested on mice or tumor models, but non reached a clinical level. Some FDA-approved nucleic acid therapies are already used for rare disease treatment, but until now, no nucleic acid had been approved for cancer treatment. Some antisense oligonucleotides have reached phase 3 trials but have not been approved, and siRNAs have not yet gone beyond phase 2. The delivery system is crucial in improving the selectivity of nucleic acids and there are already some clinical examples based on *N*-acetyl galactosamine conjugation and lipid nanoparticles with progressive results (Yamakawa et al., 2019). There are, for example reports of vectorized siRNAs in combination with classical gemcitabine treatment. The combination showed potential efficacy in pancreatic ductal adenocarcinoma patients in early-phase trials, but no more progress has been reported (Schultheis et al., 2020; Kulkarni et al., 2021). To expedite clinical emergence of combinations therapy, it is necessary to use carriers composed of FDA-approved materials, which often include lipids and a variety of biodegradable polymers. Within these constraints, scientists have devised unique formulations that allow both high drug loading capacity and co-delivery of diverse combinations of drugs. Nevertheless, due to manufacturing and regulatory issues, this approach of combined therapeutics in the same nanocarrier is still devoted to preclinical study.

There is a considerable opportunity of developing new chemotherapy-nucleic acid combinations that would extend to give better contributions to cancer therapeutics, providing well-effective, tolerated, tailored medicine, which hopefully can be implemented in oncology practice in upcoming years.

## Declaration of Competing Interest

The authors confirm that there are no known conflicts of interest associated with this publication, and there has been no significant financial support for this work that could have influenced its outcome.
